# A GABAergic system in atrioventricular node pacemaker cells controls electrical conduction between the atria and ventricles

**DOI:** 10.1038/s41422-024-00980-x

**Published:** 2024-06-07

**Authors:** Dandan Liang, Liping Zhou, Huixing Zhou, Fulei Zhang, Guojian Fang, Junwei Leng, Yahan Wu, Yuemei Zhang, Anqi Yang, Yi Liu, Yi-Han Chen

**Affiliations:** 1 State Key Laboratory of Cardiovascular Diseases, Shanghai East Hospital, School of Medicine, Tongji University, Shanghai, China; 2 Shanghai Arrhythmia Research Center, Shanghai East Hospital, School of Medicine, Tongji University, Shanghai, China; 3 Department of Cardiology, Shanghai East Hospital, School of Medicine, Tongji University, Shanghai, China; 4 Research Units of Origin and Regulation of Heart Rhythm, Chinese Academy of Medical Sciences, Shanghai, China; 5 Clinical Center for Heart Disease Research, Tongji University, Shanghai, China

**Keywords:** Mechanisms of disease, Molecular biology

## Abstract

Physiologically, the atria contract first, followed by the ventricles, which is the prerequisite for normal blood circulation. The above phenomenon of atrioventricular sequential contraction results from the characteristically slow conduction of electrical excitation of the atrioventricular node (AVN) between the atria and the ventricles. However, it is not clear what controls the conduction of electrical excitation within AVNs. Here, we find that AVN pacemaker cells (AVNPCs) possess an intact intrinsic GABAergic system, which plays a key role in electrical conduction from the atria to the ventricles. First, along with the discovery of abundant GABA-containing vesicles under the surface membranes of AVNPCs, key elements of the GABAergic system, including GABA metabolic enzymes, GABA receptors, and GABA transporters, were identified in AVNPCs. Second, GABA synchronously elicited GABA-gated currents in AVNPCs, which significantly weakened the excitability of AVNPCs. Third, the key molecular elements of the GABAergic system markedly modulated the conductivity of electrical excitation in the AVN. Fourth, GABA_A_ receptor deficiency in AVNPCs accelerated atrioventricular conduction, which impaired the AVN’s protective potential against rapid ventricular frequency responses, increased susceptibility to lethal ventricular arrhythmias, and decreased the cardiac contractile function. Finally, interventions targeting the GABAergic system effectively prevented the occurrence and development of atrioventricular block. In summary, the endogenous GABAergic system in AVNPCs determines the slow conduction of electrical excitation within AVNs, thereby ensuring sequential atrioventricular contraction. The endogenous GABAergic system shows promise as a novel intervention target for cardiac arrhythmias.

## Introduction

Without the electrical excitation of the cardiomyocytes, there is no contractile activity of the heart.^1,2^ Physiologically, electrical excitation initiates from the sinoatrial node, the headquarter of electrical activity, which first travels to the atria and then to the ventricles via the atrioventricular node (AVN) and His-Purkinje network, ultimately triggering the ventricles to contract.^2–7^ In the heart, the conduction velocity of electrical excitation within the AVN is extremely slow,^8,9^ a feature that ensures sequential atrioventricular contraction and efficient cardiac pumping.^10–12^ Clinically, AVN conduction defects are one of the most common arrhythmias, leading to varying degrees of the atrioventricular blocks (AV blocks), and even cardiac arrest and sudden cardiac death. Severe AV block is the leading clinical indication for cardiac pacemaker implantation.^13,14^ Unfortunately, little is known about the underlying mechanism of AVN dysfunction-related diseases. The phenomenon of atrioventricular conduction delay mentioned above is the electrophysiological basis for maintaining atrioventricular sequential contraction and normal contractile function of the heart, and it is of great theoretical and practical value to elucidate the mechanism of this phenomenon.

AVN pacemaker cells (AVNPCs), the main functional cells in the AVN, have neuron-like electrophysiological properties, such as excitability, conductivity, refractoriness, and automaticity, suggesting that AVNPCs share key biological characteristics with neurons.^15^ Neurons are largely dependent on transmitters to conduct electrical excitation.^16–18^ There are three types of neurotransmitters: inhibitory, excitatory, and modulatory, among which inhibitory neurotransmitters can inhibit the electrical excitation of neurons, thereby decreasing the conduction velocity of the excitation between neurons.^19–21^ Given that AVNPCs have similar biological characteristics to neurons and that the conduction velocity of electrical excitation in the AVN is unusually slow, we hypothesized that AVNPCs have their own functional inhibitory transmitters that control the electrical conduction of AVNs. GABA is a major inhibitory neurotransmitter in the central nervous system.^20,21^ However, its biological function is largely unknown in non-neural tissues,^22^ especially in the heart.

In the present study, we identified various components of the intrinsic GABAergic system in AVNPCs, including abundant GABA-containing vesicles under the surface membranes of AVNPCs and key molecular elements of the GABAergic system, such as GABA metabolic enzymes, GABA receptors, and GABA transporters. By whole-cell patch clamp recordings, we found that GABA can induce significant inward currents in single AVNPCs, suggesting that GABA is critically involved in the regulation of the electrophysiological function of the AVN. The electrophysiological examination confirmed that the GABAergic system critically modulates the excitability of AVNPCs and the conduction of electrical excitation in the AVN. In addition, GABA_A_ receptor (GABA_A_R) deficiency led to the dysfunction of AVN conduction, increasing the susceptibility to severe arrhythmias in mice. Moreover, defects in this receptor subunit also accelerated atrioventricular conduction, breaking the physiological atrioventricular conduction delay, and ultimately impairing the contractile function of the ventricle. Importantly, interventions targeting the GABAergic system significantly prevented the occurrence and development of severe AV blocks.

## Results

### Identification of an intact endogenous GABAergic system in AVNPCs

**Fig. 1:**
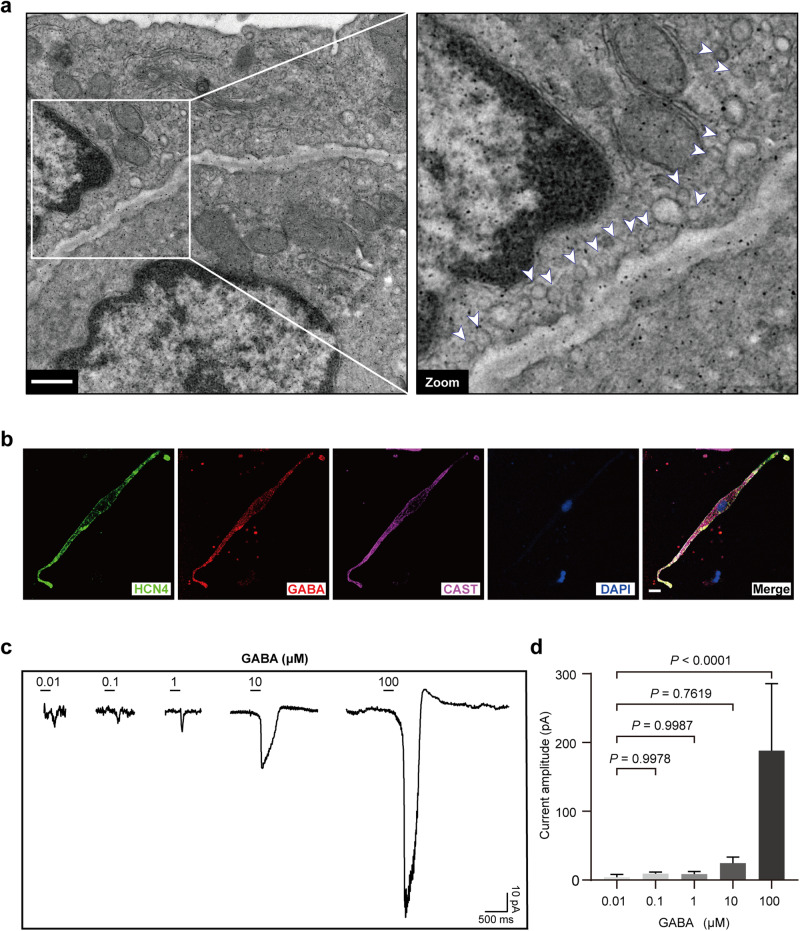
Observation of GABA-containing vesicles and GABA-induced currents in rat AVNPCs. **a** Representative TEM image showing abundant transmitter vesicle-like ultrastructures beneath the surface membranes of rat AVNPCs. Right, a magnified version of the white box in the left image. White arrows indicate the vesicles. Scale bar, 500 nm. **b** Immunofluorescence images showing the colocalization of GABA with the vesicle marker CAST in single rat AVNPCs. Scale bar, 10 μm. **c** Representative patch clamp recordings of whole-cell currents in rat AVNPCs elicited by various concentrations of GABA (0.01 μM, 0.1 μM, 1 μM, 10 μM, 100 μM). The holding potential was set at –60 mV. Horizontal bar, 500 ms; vertical bar, 10 pA. **d** Quantification of the GABA-elicited currents amplitude with different concentrations of GABA. *n* = 8 cells for each column. Data are shown as the means ± SD. *P* values were calculated by one-way ANOVA with Dunnett’s multiple comparison test.

As mentioned above, commonalities in the electrophysiological properties of AVNPCs and inhibitory neurons led us to speculate that AVNPCs may also possess an endogenous inhibitory transmitter system. First, we identified AVNs by the anatomical location and the specific immunofluorescence staining (Supplementary information, Fig. S1). Then, using transmission electron microscopy (TEM), we found abundant transmitter vesicle-like ultrastructures that were mainly located beneath the surface membrane of AVNPCs in rats (Fig. 1a). Finally, we verified the colocalization of GABA with either the vesicle marker calpastatin (CAST) or the GABA-specific vesicle transporter (vGAT) in single rat AVNPCs, indicating the existence of GABA-containing vesicles in AVNPCs (Fig. 1b; Supplementary information, Fig. S2). We demonstrated by the same techniques that these GABA-containing vesicles are also present in mouse AVNPCs (Supplementary information, Fig. S3).

Given the abundance of GABA-containing vesicles in AVNPCs, we analyzed whether GABA could act as a transmitter and elicit the ligand-gated currents in AVNPCs. Whole-cell patch clamp recordings showed that administration of GABA by spraying onto the surface membranes of isolated rat AVNPCs indeed evoked transient inward currents in a concentration-dependent manner after setting the holding potential to –60 mV (Fig. 1c, d). These results suggest that GABA has electrophysiological function in AVNPCs, similar to that in GABAergic neurons.

**Fig. 2:**
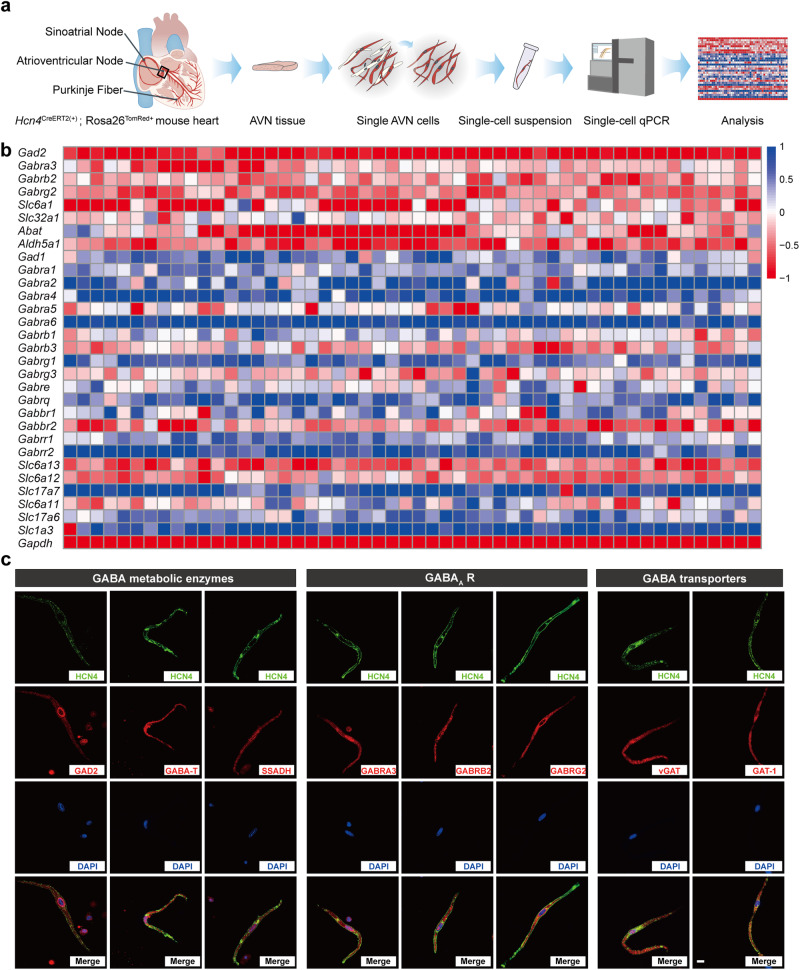
Identification of the endogenous GABAergic system in AVNPCs. **a** Illustrator and workflow for the single-cell gene expression analysis of AVNPCs from *Hcn4*^CreERT2(+)^; *Rosa26*^TomRed+^mice. **b** Heatmap showing GABAergic system gene expression in single AVNPCs from *Hcn4*^CreERT2(+)^; *Rosa26*^TomRed+^ mice. Rows indicate the AVNPC samples, and columns indicate the cycle threshold (Ct) values of GABAergic system genes. Ct values were scaled and the relative gene expression was demonstrated using color scales. A score of –1 (red) indicates a high expression level, and a score of 1 (blue) indicates a low level of expression. The data were obtained from 52 AVNPCs from 5 *Hcn4*^CreERT2(+)^; *Rosa26*^TomRed+^ mice. **c** Immunofluorescence staining showing the expression and localization of GABA metabolic enzymes (GAD2, GABA-T, and SSADH), GABA_A_ receptors (GABRA3, GABRB2, and GABRG2), GABA transporters (vGAT and GAT-1) in single mouse AVNPCs. Scale bar, 10 μm.

In the mammalian central nervous system, GABA is mainly synthesized by glutamate decarboxylases (GADs) intracellularly, loaded into vesicles by vesicular GABA transporters (vGATs), released via vesicular exocytosis into the extracellular space, then uptaken by GABA transporters (GATs) and cleared by GABA-degrading enzyme.^23^ GABA acts on either ionotropic or metabotropic receptors to ultimately regulate the electrical activity of neurons. These GABA-related metabolic enzymes, transporters, and receptors constitute a complete GABAergic system.^24^ However, the functional existence of the GABAergic system outside the brain is still not well understood. To investigate the gene expression of GABAergic system in AVNPCs, we established a tamoxifen-inducible *Hcn4*^CreERT2(+)^; *Rosa26*^TomRed+^ mouse line to obtain fluorescence-labeled AVNPCs by crossing *Hcn4*^CreERT2(+)^ mouse with *Rosa26*^TomRed+^ reporter mouse (Supplementary information, Fig. S4a). The hyperpolarization-activated cyclic nucleotide-gated cation channel HCN4 is a marker of the cardiac conduction system, particularly of the sinoatrial node and AVN. The significant expression of TomRed was observed in the cardiac conduction system of *Hcn4*^CreERT2(+)^; *Rosa26*^TomRed+^ mice, indicating the successful Cre recombination (Supplementary information, Fig. S4b). Single HCN4^*+*^ AVNPCs were isolated from heart tissues in the Koch triangle of adult *Hcn4*^CreERT2(+)^; *Rosa26*^TomRed+^ mice for gene expression analysis (Fig. 2a). The single-cell qPCR was performed to examine the expression of genes encoding key elements of the GABAergic system in AVNPCs. We found that genes encoding GABA synthetase (*Gad2*), GABA_A_R subunits (*Gabra3*, *Gabrb2*, *Gabrg2*), GABA transporters (*Slc32a1*, *Slc6a1*), GABA transferase (*Abat*), and GABA degrading enzyme (*Aldh5a1*) were highly expressed in AVNPCs (Fig. 2b).

Furthermore, immunofluorescence experiments confirmed the protein expression of these key genes of the GABAergic system in AVN tissue sections and single AVNPCs. As shown in Fig. 2c and Supplementary information, Figs. S5–S7, GAD2 (encoded by *Gad2*), GABA_A_R α3 subunit (GABRA3, encoded by *Gabra3*), GABA_A_R β2 subunit (GABRB2, encoded by *Gabrb2*), GABA_A_R γ2 subunit (GABRG2, encoded by *Gabrg2*), vesicular GABA transporter (vGAT, encoded by *Slc32a1*), GABA transporter 1 (GAT-1, encoded by *Slc6a1*), GABA transferase (GABA-T, encoded by *Abat*) and GABA degradation enzyme (SSADH, encoded by *Aldh5a1*) were abundantly expressed in AVN tissues and AVNPCs of mouse and rat. Notably, GABA_A_R subunits, including GABRA3, GABRB2, and GABRG2, were enriched in the surface membranes of AVNPCs. Collectively, we identified an intact endogenous GABAergic system in AVNPCs.

### The GABAergic system modulates the excitability of AVNPCs

**Fig. 3:**
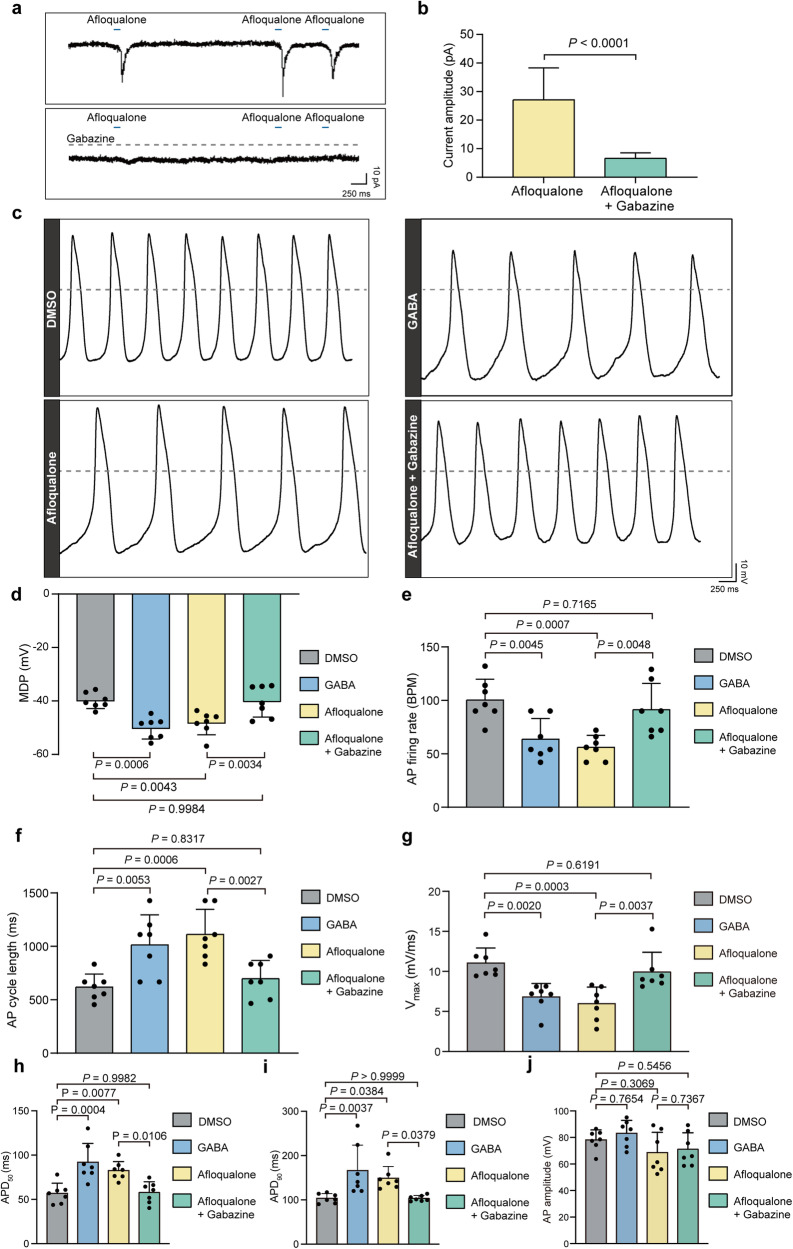
GABA and GABA_A_R activation decreases the excitability of AVNPCs. **a**, **b** Representative traces and pooled data showing the currents evoked by GABA_A_R agonist (Afloqualone, 100 μM) in rat AVNPCs (top). Afloqualone-elicited currents were blocked by of GABA_A_R antagonist (Gabazine, 30 μM) (bottom). Data are shown as the means ± SD. *n* = 13 cells for Afloqualone-treated group, *n* = 8 cells for Afloqualone + Gabazine-treated group. *P* values were calculated using two-tailed unpaired Student’s *t*-test. **c** Representative spontaneous APs recorded using current patch clamp in rat AVNPCs. The cells were treated with GABA_A_R agonist (GABA, 100 μM; Afloqualone, 100 μM), or the Afloqualone + GABA_A_R antagonists (Gabazine, 30 μM). The horizontal line marks 0 mV. Scale bars are 250 ms (horizontal) and 10 mV (vertical). **d**–**j** Pooled data of MDP, spontaneous AP firing rate, AP cycle length, max upstroke velocity (V_max_), AP duration measured at 50% (APD_50_) and 90% (APD_90_) repolarization, and AP amplitude from AP recording using current patch clamp in rat AVNPCs. *n* = 7 cells per group. Data are shown as means ± SD. *P* values were calculated by one-way ANOVA with Dunnett’s multiple comparison test.

Since electrical excitability is a prerequisite for conductivity, we first examined the effect of GABAergic system on ligand-gated currents and spontaneous action potentials (APs) in single rat AVNPCs. Given that GABA_A_R elicits the fastest inhibitory activity among mammalian neurons,^20,25,26^ and its highly abundant expression and membrane localization characteristics in AVNPCs as abovementioned, we used the GABA_A_R-specific agonist Afloqualone to detect GABA_A_R-mediated currents in AVNPCs. The results showed that a transient inward current was evoked under the clamp voltage of –60 mV (Fig. 3a, b), similar to the effect of GABA on AVNPCs. In addition, the currents induced by Afloqualone could be blocked by Gabazine, a specific antagonist for GABA_A_R (Fig. 3a, b). Spontaneous APs of AVNPCs were then recorded in current-clamp mode. The results showed that the administration of GABA or Afloqualone hyperpolarized the maximum diastolic potential (MDP), suggesting that GABA or the activation of GABA_A_R increased the electrical excitation threshold of AVNPCs (Fig. 3c, d). We also investigated whether the GABA_A_R antagonist could rescue the inhibitory effect of GABA_A_R activation on the excitability of AVNPCs. As demonstrated in Fig. 3c, d, Gabazine successfully inhibited the hyperpolarization of MDP induced by Afloqualone in AVNPCs. Moreover, we noted that the administration of GABA or Afloqualone resulted in a reduction of spontaneous AP firing rate, and an elongation of the spontaneous AP cycle length, a diminished max upstroke velocity, and an extension of AP duration measured at 50% (APD_50_) and 90% (APD_90_) repolarization periods (Fig. 3e–i). These changes caused by Afloqualone could be attenuated by Gabazine (Fig. 3c–i). There was no significant change in AP amplitude in different groups (Fig. 3j). These data indicated the critical regulatory effect of GABA and GABA_A_R on the excitability of AVNPCs.

### The GABAergic system controls the conduction of electrical excitation within the AVN

**Fig. 4:**
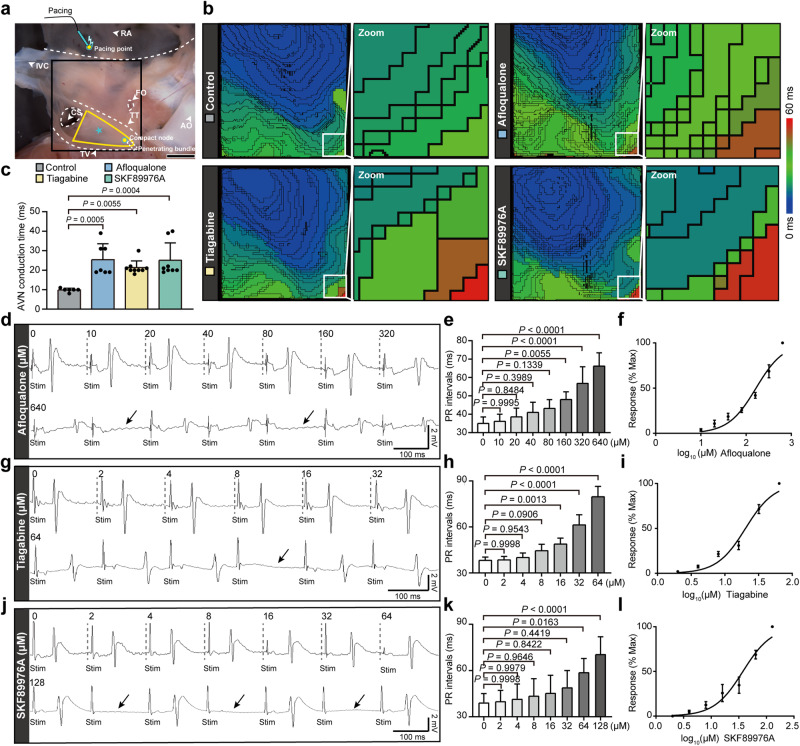
The GABAergic system controls electrical conduction within the AVN. **a** Photograph of isolated rat AVN tissue and optical mapping of the field of view. The pacing electrode is marked with a blue oval. The black box shows the electrical signal mapping field. The yellow triangle shows the position of the Koch triangle. Anatomical landmarks are annotated. FO fossa ovalis, TV tricuspid valve, TT tendon of Todaro, CS coronary sinus, RA right atrium, IVC inferior vena cava, AO aorta. Scale bar, 2 mm. **b** Representative activation map showing the electrical activation and conduction in isolated rat AVN preparations after perfusion with the solvent control DMSO, the GABA_A_R-specific agonist Afloqualone (320 μM), the GABA reuptake inhibitor Tiagabine (64 μM) or the GAT-1 inhibitor SKF89976A (128 μM). The activation map of zoomed-in images shows the electrical activation and conduction in the compact node of the AVN. Electrical activity in rat AVN preparations was recorded by optical mapping using the fluorescent dye Di-4-ANBDQBS. The activation time, conduction velocity, and vector maps were obtained during continuous electrode pacing located at the crista terminalis (5 Hz, 2 V). **c** Quantification of electrical conduction time within the AVN in distinct groups (*n* = 6 samples for control group, *n* = 7 samples for Afloqualone-treated group, *n* = 9 samples for Tiagabine-treated group, *n* = 8 samples for SKF89976A-treated group). Data are shown as means ± SD. *P* values were calculated by one-way ANOVA with Dunnett’s multiple comparison test. **d**, **g**, **j** Representative ECG recordings of perfused rat hearts treated with different concentrations of Afloqualone (0–640 μM) (**d**), Tiagabine (0–64 μM) (**g**) or SKF89976A (0–128 μM) (**j**) under the right atrial pacing (6 Hz). The arrows point to the typical ECG of second-degree type I AV block (**d**, **g**) and second-degree AV block (2:1) (**j**). Scale bars are 100 ms (horizontal) and 2 mV (vertical). Stim stimulation. **e**, **h**, **k** The dose-response of Afloqualone (**e**), Tiagabine (**h**) and SKF89976A (**k**) on PR intervals in perfused rat hearts. *n* = 5 hearts for Afloqualone and SKF89976A treatment group, *n* = 6 hearts for Tiagabine-treated group. Data are shown as means ± SD. *P* values were calculated using one-way ANOVA with Dunnett’s multiple comparison test. **f**, **i**, **l** Concentration-response curves of PR interval alterations induced by Afloqualone (EC_50_, 165.70 μM) (**f**), Tiagabine (IC_50_, 20.22 μM) (**i**), and SKF89976A (IC_50_, 37.57 μM) (**l**). Concentration-response curves were fitted to the Hill equation using nonlinear regression and normalized to the maximal changed PR intervals. Data are shown as means ± SD. *n* = 5 hearts for Afloqualone- and SKF89976A-treated group, *n* = 6 hearts for Tiagabine-treated group.

Considering that GABAergic system can regulate the excitability of AVNPCs, it is possible that this system can regulate the conductivity of electric excitation between these pacemaker cells. Next, we analyzed the effects of the GABAergic system on electrical conductivity in rat AVN tissues. Initially, we evaluated the influence of GABA_A_R activation on AVN conduction velocity using optical mapping in the context of right atrial pacing (Fig. 4a). As shown in Fig. 4b, c, the conduction time significantly increased within AVN tissues treated with the GABA_A_R agonist Afloqualone compared with the control group, suggesting that the activation of GABA_A_R reduced the electrical conduction velocity of the AVN. Furthermore, we examined the effect of GABA and GATs on the conductivity of the AVN. In the brain, the uptake of GABA from the synaptic cleft into the cell is mediated by GATs.^26–29^ The suppression of GABA uptake processes increases extracellular GABA amount and tonic inhibition.^30^ We found that the administration of either the GABA reuptake inhibitor (Tiagabine) or the GAT-1 inhibitor (SKF89976A) also decreased the electrical conduction velocity of AVN tissues (Fig. 4b, c). Collectively, our results suggested that as key elements of the GABAergic system, GABA_A_R and GABA transporter modulate the conduction of electrical excitation within the AVN tissue.

We further investigated the role of the GABAergic system in atrioventricular electrical conduction, which was evaluated based on the PR intervals of electrocardiogram (ECG) in perfused rat hearts. During the experiments, right atrial pacing was performed to control the beating rates of the perfused rat hearts, which subtracted the effect of heart rates on the PR intervals. The results showed that the GABA_A_R agonist Afloqualone caused the prolongation of PR intervals in a concentration-dependent manner (Fig. 4d–f). The PR intervals were extended from 35.00 ± 3.54 ms to 66.20 ± 7.19 ms (0–640 μM) after Afloqualone treatment (Fig. 4d–f). The half maximal effective concentration (EC_50_) was 165.70 μM (95% confidence interval (CI) = 140.90, 194.90 μM). Notably, the hearts even undergone cardiac arrest when perfused with Afloqualone at 640 μM due to severe conduction block (Supplementary information, Fig. S8a). We also evaluated the effects of the GABA reuptake inhibitor Tiagabine and the GAT-1 inhibitor SKF89976A on PR intervals and found that either Tiagabine or SKF89976A caused a concentration-dependent prolongation of PR intervals in isolated rat hearts. The PR intervals were extended from 38.17 ± 2.23 ms to 79.67 ± 6.83 ms (0–64 μM) after perfusion with Tiagabine (Fig. 4g–i). SKF89976A prolonged the PR intervals from 39.40 ± 5.68 ms to 70.80 ± 11.08 ms (0–128 μM) (Fig. 4j–l). The half maximal inhibitory concentrations (IC_50_) were 20.22 μM (95% CI = 18.10, 22.59 μM, Tiagabine) and 37.57 μM (95% CI = 31.05, 45.45 μM, SKF89976A), respectively. We observed that the high concentration of Tiagabine (64 μM) or SKF89976A (128 μM) could cause second- and even third-degree AV block (Fig. 4g, j; Supplementary information, Fig. S8b, c). In addition, Afloqualone, Tiagabine, and SKF89976A did not alter QRS durations or QT intervals in perfused rat hearts (Supplementary information, Fig. S9).

### The knockout of *Gabrb2* markedly accelerates the electrical conduction within the AVN and drastically reduces the AVN’s ability to protect against excessive ventricular frequency response in mice

**Fig. 5:**
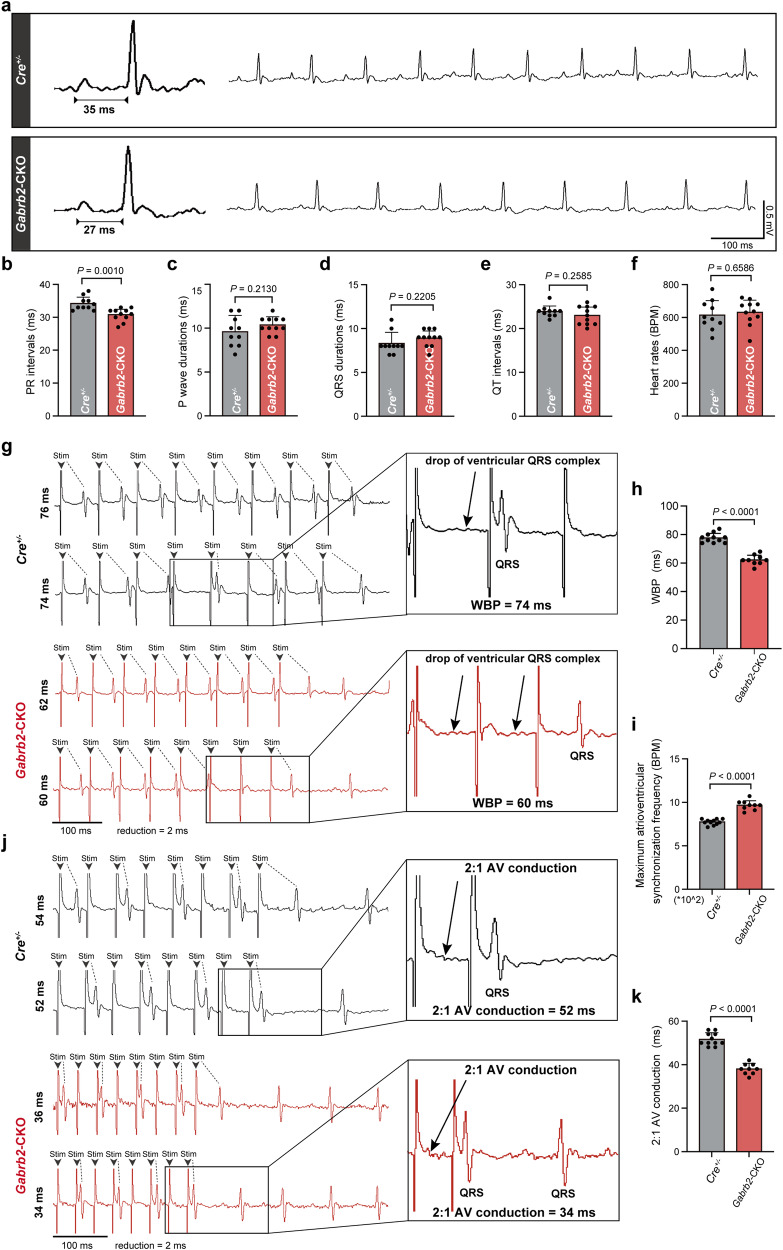
*Gabrb2* knockout accelerates the electrical conduction between the atria and the ventricles in vivo. **a** Representative ECG traces obtained from conscious *Cre*^+/−^ and *Gabrb2*-CKO mice using telemetric recording system. Scale bars are 100 ms (horizontal) and 0.5 mV (vertical). **b**–**f** Quantification of the average PR intervals (**b**), P wave durations (**c**), QRS durations (**d**), QT intervals (**e**) and heart rates (**f**) of mice in the indicated groups. BPM beats per minute. Data are shown as means ± SD. *P* values were calculated using two-tailed unpaired student’s *t*-test. *n* = 10 mice for *Cre*^+/−^ group and *n* = 11 mice for *Gabrb2*-CKO group. **g**–**k** Intracardiac programmed electrical stimulation (PES) was used to examine the electrical physiological function parameters of AVN including WBP (**g**, **h**), maximum atrioventricular synchronization frequency (**i**) and 2:1 AV conduction (**j**, **k**) from *Cre*^+/−^ and *Gabrb2*-CKO mice. **g**, **j** Representative ECG traces for evaluation of the above parameters by PES. The drop of ventricular QRS complex pointed by the arrows is highlighted in the black box. The 2:1 AV conduction indicates the Wenckebach point that only one QRS complex was generated by two S1 stimulations. Data are shown as means ± SD. *P* values were calculated using two-tailed unpaired student’s *t*-test. *n* = 11 mice for *Cre*^*+*^ group and *n* = 9 mice for *Gabrb2*-CKO group. Stim stimulation.

**Fig. 6:**
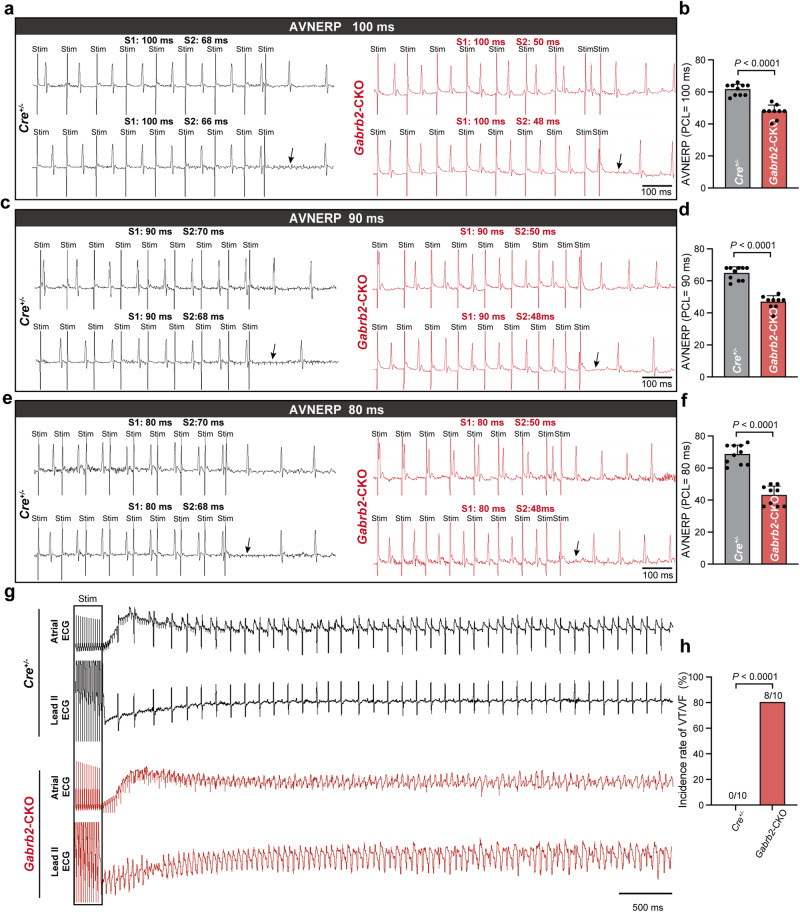
*Gabrb2* knockout increases the susceptibility to fatal ventricular arrhythmia in mice. **a**–**f** Representative images and quantification of AVNERP. The AVNERP was evaluated by PES under different PCLs in *Cre*^+/−^ mice and *Gabrb2*-CKO mice. The short trains of eight atrial stimuli (S1) followed by 1 extrastimulus (S2) were applied. Arrows indicate loss of S2-induced ventricular signal. Data are shown as means ± SD. *P* values were calculated using two-tailed unpaired student’s *t*-test. *n* = 10 mice for *Cre*^+/−^ group and *n* = 9 mice for *Gabrb2*-CKO group. **g**, **h** Representative intracardiac ECG traces and quantification of the occurrence of fatal ventricular arrhythmias from *Cre*^+/−^ and *Gabrb2*-CKO mice after the challenge of isoproterenol and high frequency atrial burst stimulation. Atrial ECG represents the ECG signals from right atrium; Lead II ECG represents the surface ECG recordings. *n* = 10 mice per group. *P* values were calculated by Fisher’s exact test. VT ventricular tachycardia, VF ventricular fibrillation, Stim stimulation.

As demonstrated above, activating or inhibiting key components of the GABAergic system can significantly alter the conduction of electrical excitation in the AVN. Next, in order to further analyze the regulatory effect of the GABAergic system on the conduction of electrical excitation, we conditionally deleted *Gabrb2* encoding GABRB2, a crucial component of the GABAergic system, within the cardiac conduction system by crossing *Gabrb2*^flox*/*flox^ mice with *Hcn4*^CreERT2(+)^ (*Cre*^+/−^) mice (Supplementary information, Fig. S10a). The expression of *Gabrb2* was significantly decreased in the AVN of *Gabrb2*^flox*/*flox^ × *Cre*^+/−^ (*Gabrb2*-CKO) mice, indicating successful deletion of the *Gabrb2*^flox*/*flox^ allele in AVNPCs (Supplementary information, Fig. S10b). As expected, we observed a significantly shortened PR intervals in *Gabrb2*-CKO mice (30.82 ± 1.94 ms) compared with *Cre*^+/−^ mice (34.20 ± 2.04 ms) at 10 weeks after tamoxifen induction (Fig. 5a, b), indicating its role in regulating atrioventricular electrical conduction. Other ECG parameters including the P wave durations, QRS durations, QT intervals and heart rates were not altered in *Gabrb2*-CKO mice (Fig. 5c–f). Considering that the excitability of cells determines the conductivity of electrical excitation between them, we investigated the changes in excitability of AVNPCs after the knockout of *Gabrb2*. The whole-cell patch clamp examinations showed that *Gabrb2* knockout depolarized MDP, indicating that *Gabrb2* knockout decreased the electrical excitation threshold of AVNPCs. At the same time, we found that the knockout of *Gabrb2* led to an elevated spontaneous AP firing rate, a reduced spontaneous AP cycle length, an enhanced max upstroke velocity, and a decrease in the duration of APD_50_ and APD_90_ repolarization periods (Supplementary information, Fig. S11a–g). There was no difference in AP amplitude between the two groups (Supplementary information, Fig. S11h). The slow electrical conduction properties of the AVN can reduce the delivery of supraventricular electrical excitation at excessively high frequencies to the ventricles, thus preventing an overly rapid ventricular frequency response.^10^ Based on the significant effects of *Gabrb2* on AVN conductivity, we further explored its potential role in ventricular rate maintenance. Intracardiac electrophysiological recordings were performed to evaluate the electrical conduction function of AVN after *Gabrb2* knockout. The results demonstrated a significant reduction in Wenckebach periodicity (WBP) in *Gabrb2*-CKO mice (62.22 ± 3.53 ms) when compared with *Cre*^+/−^ mice (77.64 ± 3.33 ms) (Fig. 5g, h). Meanwhile, the maximum atrioventricular synchronization frequency was significantly increased from 774.10 ± 32.47 bpm in *Cre*^+/−^ mice to 967.10 ± 55.31 bpm in *Gabrb2*-CKO mice (Fig. 5i). In addition, in vivo programmed electrical stimulation demonstrated a decreased 2:1 atrioventricular conduction (2:1 AV conduction), which displayed a shorter atrial burst pacing cycle length (PCL) in *Gabrb2*-CKO mice (38.00 ± 2.45 ms) compared with *Cre*^+/−^ mice (51.64 ± 2.94 ms) (Fig. 5j, k). Then, through intracardiac programmed electrical stimulation, we examined the AVN effective refractory period (AVNERP), which can reflect the conductivity of electrical excitation within AVN. A notably reduced AVNERP was observed in *Gabrb2*-CKO mice compared with *Cre*^+/−^ mice (Fig. 6a–f). Together, these findings demonstrate a marked acceleration in AVN conductibility following *Gabrb2* knockout.

To avoid the influence of autonomic nerves system activity on the electrical conductivity of AVN, we tested the above parameters that reflect the conduction properties of the AVN in *Cre*^+/−^ mice and *Gabrb2*-CKO mice subjected to Langendorff ex vivo heart perfusion. The results showed that the hearts of *Gabrb2*-CKO mice presented lower WBP, higher maximum atrioventricular synchronization frequency, more enhanced 2:1 AV conduction and shorter AVNERP when compared with those of *Cre*^+/−^ mice, which was consistent with the results from in vivo experiments (Supplementary information, Fig. S12).

To further elucidate the role of *Gabrb2* in the ventricular response to high frequency, we conducted functional rescue experiments using the GABA_A_R-specific agonist Afloqualone. Intracardiac electrophysiological recordings were performed in *Cre*^+/−^ mice after intracardiac administration of Afloqualone (320 μM). The results showed that in mice treated with Afloqualone, there was a significant increase in WBP, a reduction in maximum atrioventricular synchronization frequency, a diminishment in 2:1 AV conduction, and a prolongation of AVNERP compared with the control group (Supplementary information, Figs. S13, S14). These results highlight Afloqualone’s impact on the electrical conduction of the AVN. We then performed intracardiac electrophysiological recordings in *Gabrb2*-CKO mice with intracardiac treatment of Afloqualone (320 μM). We also observed an increase in WBP, a reduction in maximum atrioventricular synchronization frequency, a diminishment in 2:1 AV conduction, and a prolongation of AVNERP in Afloqualone-treated *Gabrb2*-CKO mice compared with DMSO-treated *Gabrb2*-CKO mice. These data indicated that Afloqualone can attenuate the effect of *Gabrb2* knockout on AVN electrical conduction, which further clarify the role of *Gabrb2* in the electrical conduction within the AVN (Supplementary information, Figs. S15, S16).

### The knockout of *Gabrb2* increases susceptibility to fatal ventricular arrhythmias and impairs ventricular contractile function

The data above indicate that *Gabrb2* knockout greatly enhances the conductivity of the AVN, consequently significantly reducing its protective potential against excessive ventricular frequency response. After the crucial protective capacity of the AVN is impaired, rapid supraventricular electrical excitation is more likely to fall into the vulnerable period of the ventricular myocardium, thereby triggering fatal ventricular arrhythmias. In light of this, by intracardiac ECG recordings, we tested the susceptibility of *Gabrb2*-CKO mice to ventricular tachycardia/ventricular fibrillation (VT/VF). We observed a significantly higher incidence of VT/VF in *Gabrb2*-CKO mice compared with *Cre*^+/−^ mice following high-frequency atrial burst stimulation after isoproterenol induction (Fig. 6g, h). This suggests that faster atrioventricular conduction due to *Gabrb2* knockout may lead to fatal ventricular arrhythmias.

It is widely recognized that a delay in atrioventricular conduction is essential for ensuring adequate ventricular diastole, which in turn facilitates the optimal filling of the ventricles with blood from the atria, thus preserving normal cardiac contractility.^15,31^ Based on this knowledge of physiology and pathophysiology, we tracked the effects of *Gabrb2* deficiency on ventricular contractile function. We found that the ejection fraction (EF%) and fractional shortening (FS%) were significantly decreased in *Gabrb2*-CKO mice compared with *Cre*^+/−^ mice (Supplementary information, Fig. S17), suggesting that *Gabrb2* knockout impaired cardiac contractile function. Taken together, when elements of the GABAergic system such as the GABA_A_R are defective, the AVN partially loses its protective role in maintaining ventricular systolic function under pathological conditions.

### Key elements of GABAergic system act as potential intervention targets for AV blocks

**Fig. 7:**
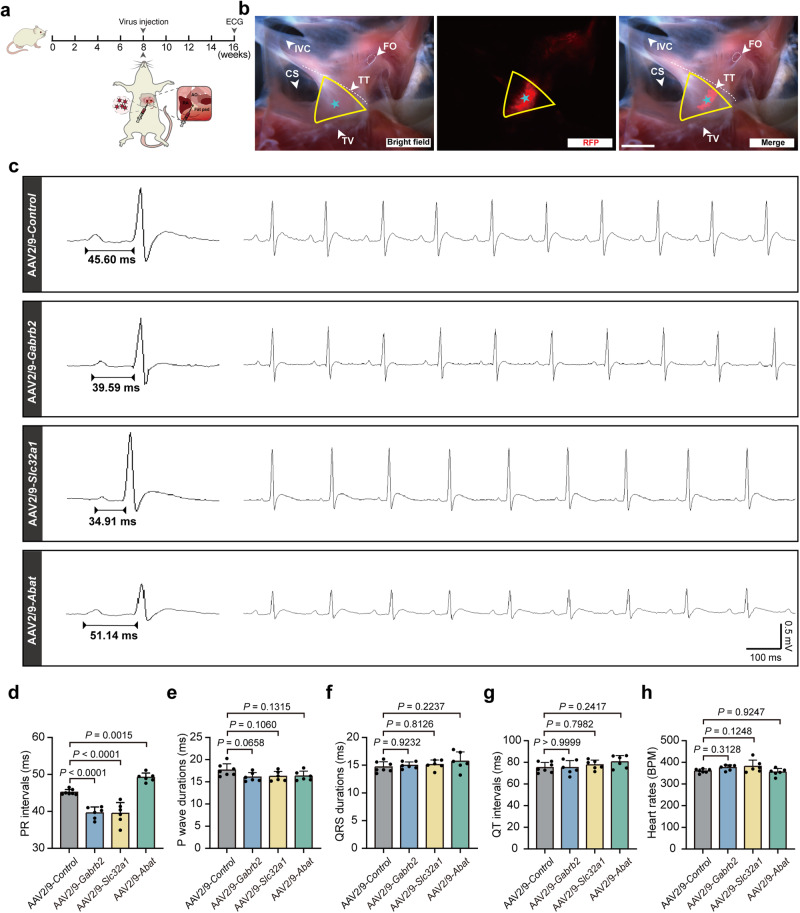
Knockdown of key genes in GABAergic system alters electrical conduction between the atria and ventricles in vivo. **a** Top, a cartoon illustration of experimental design. Bottom, schematic diagram of local injection of virus into rat AVN. AO aorta, RA right atrium. **b** Fluorescence microscopy confirmed that RFP was highly expressed in Koch’s triangle injected with AAV2/9 virus. Bright-field (left), RFP (middle) and merged images (right) of hearts injected with AAV2/9 virus. The yellow triangle indicates the triangle of Koch area. The asterisk denotes AVN location. Scale bar, 2 mm. FO fossa ovalis, TV tricuspid valve, TT tendon of Todaro, CS coronary sinus, IVC inferior vena cava. **c** Representative ECGs obtained from conscious rats through a telemetric recording system after administration of AAV2/9-*Control*, AAV2/9-*Gabrb2*, AAV2/9-*Slc32a1* or AAV2/9-*Abat* virus. Scale bars are 100 ms (horizontal) and 0.5 mV (vertical). **d**–**h** Quantification of the average PR intervals (**d**), P wave durations (**e**), QRS durations (**f**), QT intervals (**g**) and heart rates (BPM, beats per minute) (**h**) of rats in the indicated groups. For AAV2/9-*Control* group, *n* = 7; for AAV2/9-*Gabrb2* group, *n* = 6; for AAV2/9-*Slc32a1* group, *n* = 6; for AAV2/9-*Abat* group, *n* = 6. Data are shown as means ± SD. *P* values were calculated by one-way ANOVA with Dunnett’s multiple comparison test.

To explore the capability of the GABAergic system as a potential target for arrhythmias resulting from AVN dysfunction, we developed the AAV2/9-CAG-mCherry viruses for the knockdown of genes encoding the critical components of the GABAergic system: *Gabrb2*, *Slc32a1* or *Abat*. The viruses were then respectively delivered to rat AVN tissues by localized injection using an insulin syringe (Fig. 7a). The successful infection of AAV2/9-CAG-mCherry viruses in AVN tissues was confirmed through fluorescence microscopy (Fig. 7b). The effective knockdown of *Gabrb2*, *Slc32a1*, and *Abat* was validated by qPCR and fluorescence assays in AVN tissues injected with AAV2/9-CAG-mCherry virus (Supplementary information, Figs. S18, S19). At 8 weeks after virus injection, we found that the knockdown of *Gabrb2* or *Slc32a1* shortened PR intervals, and *Abat* knockdown prolonged PR intervals in rat hearts (Fig. 7c, d). However, the knockdown of *Gabrb2*, *Slc32a1* or *Abat* did not affect the P wave durations, QRS durations, QT intervals, or heart rates (Fig. 7e–h). These data suggested that the deficiencies of key elements of the GABAergic system in AVNPCs led to conduction abnormalities between the atria and ventricles.

**Fig. 8:**
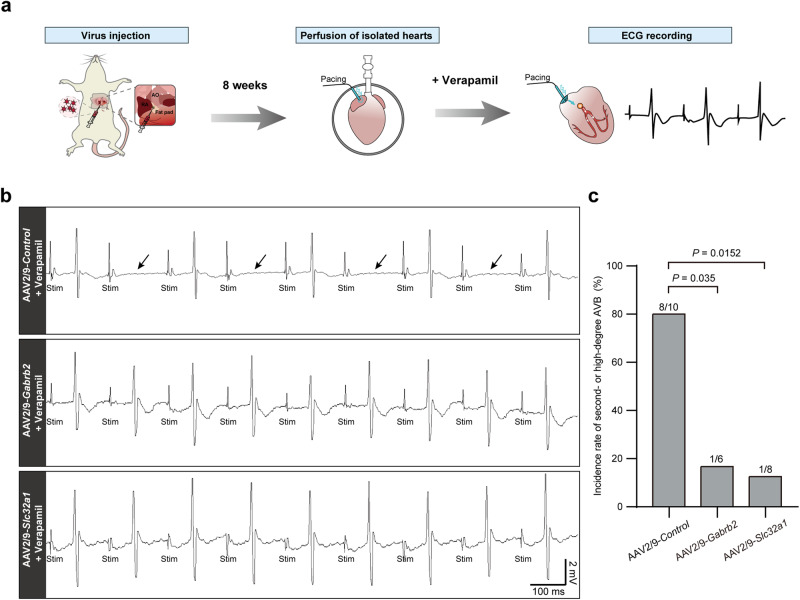
Intervention targeting the GABAergic system prevents the occurrence and development of AV block. **a** Experimental design. AO aorta, RA right atrium. **b** Representative ECG recordings showing successful construction of severe AV block by perfusion with verapamil (250 nM) in isolated hearts of AAV2/9-*Control* virus-injected rats. Arrows indicate the representative second-degree AV block (2:1). AAV2/9-*Gabrb2* or AAV2/9-*Slc32a1* virus-injected hearts did not develop second- or high-degree AV block. Stim stimulation. Scale bars are 100 ms (horizontal) and 2 mV (vertical). **c** Proportion of second- or high-degree AV block in AAV2/9-*Control*, AAV2/9-*Gabrb2* or AAV2/9-*Slc32a1* virus-injected hearts. *n* = 10 rats for AAV2/9-*Control* group, *n* = 6 rats for AAV2/9-*Gabrb2* group, and *n* = 8 rats for AAV2/9-*Slc32a1* group. *P* values were calculated by Fisher’s exact test.

As a common arrhythmia associated with AVN defects, AV blocks can be subdivided into first-, second-, and third-degree AV block according to the severity of the blockade.^13^ Considering the notable reduction in PR intervals in rat hearts following the knockdown of *Gabrb2* or *Slc32a1* (Figs. 6a, b, 7c, d), we speculated that *Gabrb2* and *Slc32a1* may be effective targets to prevent AV block occurrence or terminate AV block development by recovering normal conduction of the AVN. We then constructed an AV block model in isolated rat hearts by perfusion with 250 nM verapamil (Fig. 8a), which induced second- or high-degree AV block (Fig. 8b, c). Compared with the control group, the incidence of second- or high- degree AV block was significantly reduced in knockdown rats generated by AAV2/9-*Gabrb2* or AAV2/9-*Slc32a1* virus injection (Fig. 8b, c). These data indicate that defects in key components of the GABAergic system are associated with arrhythmia, and that the system has the potential to act as a systemic intervention target for AV blocks.

## Discussion

Atrioventricular sequential contraction relies on the delay in atrioventricular conduction, yet the underlying mechanism of this delay remains incompletely elucidated.^8^ As an important existence in the field of cardiac electrophysiology, atrioventricular conduction delay in ECG is represented by the PR intervals. Abnormal PR intervals can lead to the disruption of normal excitation and contraction of the ventricle.^32^ Our study found that AVNPCs possess an intact endogenous GABAergic system, including abundant GABA-containing vesicles under the surface membranes of AVNPCs, GABA metabolic enzymes, GABA receptors and GABA transporters, which effectively regulates the excitability of AVNPCs and the conduction of electrical excitation between the atria and the ventricles. GABA acts as a transmitter to elicit the ligand-gated currents in AVNPCs. The above effects of GABA enable the GABAergic system to effectively modulate the excitability of AVNPCs and conductivity of AVNs. We also found that GABA_A_R deficiency can significantly accelerate atrioventricular conduction, impair physiological atrioventricular conduction delay, and contribute to susceptibility to fatal ventricular arrhythmias and ventricular contractile dysfunction. Further study showed that interventions targeting the GABAergic system prevent the occurrence and development of AV blocks.

The electrical excitability and conductivity of AVNPCs are fundamental to the function of AVN. We revealed that the ionotropic GABA_A_R subtype was highly expressed in AVNPCs. The intervention targeting GABA_A_R significantly modulated the GABA-gated currents, which in turn affected the excitability of AVNPCs. In addition, we also examined the role of key GABAergic system elements in the electrical conduction of the AVN. Activating GABA_A_R, inhibiting GAT-1, or reducing GABA reuptake notably decreased the electrical conduction velocity within the AVN. These data suggest that GABAergic system exhibits an inhibitory effect on the AVN conduction and may be a new control system for electrophysiological function of the AVN.

The mechanistic explanation for the conduction delay phenomenon has always been an important topic in the field of heart rhythm. Here, we provide a series of evidence to consistently demonstrate the important role of the endogenous GABAergic system in the AVN electrical conduction. In the present study, we found that GABA_A_R deficiency in AVNPCs impaired the physiological delay of atrioventricular conduction, resulting in the increased susceptibility to fatal ventricular arrhythmias and decreased ventricular contractile function. Thus, we uncover a principle for determining the physiological slow conduction in the AVN and a unique mechanism of atrioventricular sequential contraction.

Due to the extremely limited number of AVNPCs and the complexity of electrophysiological function, the research progress on AV block therapies is lagging behind. This study found that knockdown of the GABAergic system-related gene *Gabrb2* or *Slc32a1* in AVN dramatically shortened the PR intervals in vivo, suggesting that dysregulation of GABA_A_R or vGAT may be associated with AV blocks. Importantly, we demonstrated that the knockdown of GABA_A_R or vGAT prevented the occurrence and development of severe AV blocks. GABAergic system has been shown to be a therapeutic target for various neurological diseases.^33,34^ Our findings suggest that GABAergic system and its related intervention agents have potential applications in drug development for AVN deficiency-related arrhythmias.

Recently, we found that sinoatrial node pacemaker cells share dominant biological properties with glutamatergic neurons.^35^ Further study showed that atrial cardiomyocytes also have an intrinsic glutamatergic system, which regulates the excitability and conductivity of atrial cardiomyocytes.^36^ Inhibition of ionic glutamate receptors effectively prevented and terminated the occurrence and development of atrial fibrillation.^36^ The glutamatergic system is excitatory transmitter system and mediates the fast conduction in the atria. Here, our finding sheds light on the mechanism of slow conduction within AVN from the perspective of an inhibitory transmitter system. We speculate that these inherent transmitter systems should be responsible for the different electrophysiological characteristics in distinct parts of the heart, thus maintaining the physiological heart rhythm and the normal contractile function of heart.

In summary, our research demonstrates that the intrinsic GABAergic system within AVNPCs plays a pivotal role in modulating the conductivity of the AVN, establishing the foundation for the slow electrical conduction observed between the atria and ventricles. According to traditional views, atrioventricular conduction is dependent on gap junctions and ion channels between AVNPCs and is modulated by autonomic nerves along with various other factors.^9,37–41^ Our research provides a series of evidence for both the existence and the substantial role of the intrinsic GABAergic system within the AVN. Identifying the intrinsic GABAergic system in the AVN not only deepens our understanding of the mechanisms underlying atrioventricular sequential contraction and AV blocks but also marks a significant paradigm shift in arrhythmia prevention and treatment strategies.

## Materials and methods

### Animals

All animal experiments were performed in accordance with the Guide for the Care and Use of Laboratory Animals published by the U.S. National Institutes of Health (National Academies Press; 2011) and approved by the Animal Care and Use Committee at Tongji University School of Medicine. The animals were housed (3–5 per cage) in a temperature-controlled room under a 12 h light/dark cycle with free access to water and food.

### Generation of the mouse lines

*Hcn4*^CreERT2(+)^ mice were obtained from Jackson Laboratories (Jackson Lab Stock # 024283). The *Rosa26*^TomRed+^ reporter mouse line was kindly provided by Dr. Bin Zhou (Shanghai Institutes of Biochemistry and Cell Biology). To label the AVN, we crossed *Hcn4*^CreERT2(+)^ mouse and *Rosa26*^TomRed+^ reporter mouse to generate the *Hcn4*^CreERT2(+)^; *Rosa26*^TomRed+^ mouse line. *Hcn4*^Cre*ERT2*(+)^ mice and *Rosa26*^TomRed+^ reporter mice were genotyped as previously described.^42^ The *Gabrb2*-floxed mouse line was constructed in GemPharmatech Co., Ltd. For the construction of *Gabrb2*-floxed mice, *flox* modification was performed on exon 5 of *Gabrb2* gene by using CRISPR/Cas9 technology. The cardiac conduction system-specific *Gabrb2* knockout mouse line (*Gabrb2-*CKO) was obtained by crossing *Gabrb2*^flox/flox^ mice with *Hcn4*^CreERT2(+)^ mice. The genotyping primers are provided in Supplementary information, Table S1. To induce Cre recombination and achieve fluorescence labeling of AVNPCs in *Hcn4*^CreERT2(+)^; *Rosa26*^TomRed+^ mice, and to induce cardiac conduction system-specific deletion of *Gabrb2*, tamoxifen solution (10 mg/mL, Sigma, T5648) was injected intraperitoneally (i.p.) at a dose of 80 mg/kg per day for 5 consecutive days at 8 weeks old. Age-matched male *Hcn4*^Cre*ERT2*(+)^ (*Cre*^+/−^) mice served as the controls for male *Gabrb2*-CKO mice.

### Isolation of AVNPCs

Single AVNPCs were isolated from adult *Hcn4*^CreERT2(+)^; *Rosa26*^TomRed+^ mice (10–12 weeks old), adult wild-type mice (8–12 weeks old) and adult rats (8–12 weeks old), using a modified method based on the previously reported protocol.^43–45^ Briefly, the rat or mouse was heparinized (> 200 units per animal, i.p.) and anesthetized (pentobarbital sodium, 30 mg/kg). The hearts were then quickly removed and perfused retrogradely via the aorta with warm solution A containing 140 mM NaCl, 5.4 mM KCl, 1.8 mM CaCl_2_, 1.2 mM KH_2_PO_4_, 5.5 mM D-glucose, 5 mM HEPES, with the pH adjusted to 7.4 with NaOH, followed by perfusing with solution B comprising 140 mM NaCl, 5.4 mM KCl, 0.2 mM CaCl_2_, 1.2 mM KH_2_PO4, 50 mM Taurine, 18.5 mM D-glucose, 5 mM HEPES, with the pH adjusted to 6.9 with KOH. Next, the hearts were perfused with digestion solutions containing 1 mg/mL type 2 collagenase (Worthington, LS004177), 0.01 mg/mL elastase (Sigma, E1250) and 0.6 mg/mL protease (Sigma, P5147) in solution B for 20–25 min. The perfusion solution was maintained at 37 °C and oxygenated with 100% O_2_. The enzyme concentration and digestion time were adjusted to achieve optimal digestion in different species. After digestion, the AVN tissues were cut into small pieces and transferred to solution D containing 100 mM K-glutamic acid, 10 mM K-aspartate, 25 mM KCl, 10 mM KH_2_PO_4_, 2 mM MgSO_4_, 20 mM Taurine, 5 mM Creatine, 0.5 mM EGTA, 20 mM Glucose, 5 mM HEPES and 1 mg/mL BSA, with the pH adjusted to 7.2 with KOH. Single AVNPCs were mechanically isolated by gently pipetting in solution D at 37 °C for 2–5 min. The cells were then readapted stepwise to a physiological extracellular Ca^2+^ concentration for further patch clamp recording.

### Whole-cell patch clamp recording

Whole-cell patch clamp recordings were performed on single rat AVNPCs to examine the membrane potentials and currents using an EPC-10 amplifier (HEKA, Germany) and Clampfit 10.7 software (HEKA, Germany). The membrane potentials and currents were recorded at 22–26 °C. Two types of microelectrodes (borosilicate glass, 2–6 MΩ) fabricated using a horizontal puller (Sutter Instrument) were used: one type for electrical stimulation and recording, which was filled with intracellular solution containing 140 mM KCl, 10 mM EGTA, 10 mM HEPES, 5 mM glucose, 3 mM Na_2_ATP, with the pH adjusted to 7.2 with KOH; the other type was a microelectrode with an inner diameter of 10 μm for rapid and focal application of different pharmacological agents on the membranes of AVNPCs membrane by using a PL1–100 picoinjector (Harvard Apparatus, USA), filled with an extracellular solution containing 140 mM NaCl, 5.4 mM KCl, 1.8 mM CaCl_2_, 1.2 mM KH_2_PO_4_, 50 mM Taurine, 18.5 mM D-glucose, 5 mM HEPES, 1 mg/mL BSA. The pharmacological agents (GABA, Afloqualone, and Gabazine) were dissolved in extracellular solution. For the Gabazine + Afloqualone group, AVNPCs were incubated in extracellular solution containing Gabazine (30 μM) and then Afloqualone (100 μM) was rapidly applied to AVNPCs to detect the evoked currents. Stimulation pulses were applied via an EPC-10 amplifier (HEKA, Germany) synchronized with the picoinjector to release pharmacological agents. The AP was recorded in the current-clamp mode, and the current was recorded in the voltage-clamp mode. GABA-induced current was defined as the difference between the holding current before and after the application of GABA, and the Afloqualone-induced current was defined as the difference between the holding current before and after the application of Afloqualone.

### qPCR

Single AVNPCs for RNA extraction were obtained from adult *Hcn4*^CreERT2(+)^; *Rosa26*^TomRed+^ mice (10–12 weeks old). Total RNA was extracted from single RFP-labeled AVNPCs which were picked manually under the microscope. The selected single cell was gently transferred into 1.5 μL lysis buffer. The prepared RNA was reverse transcribed to first-strand cDNA using SuperScript™ III Reverse Transcriptase (Invitrogen, 18080044). The cDNA was then amplified with KAPA HiFi HotStart ReadyMix (KAPA, KK2602), and it was quantified with a Qubit™ 4 Fluorometer (Thermo Fisher Scientific, Singapore). Subsequently, the cDNA was diluted for qPCR. Total RNA of AVN tissues from AAV2/9 virus-injected rats was isolated using GenElute Single Cell RNA Purification Kit (Sigma, RNB300). Purified RNA was reverse transcribed by the PrimeScript RT Reagent kit (Takara Bio, RR037A). For the qPCR assay, the reaction mixture (10 μL) was amplified using the SYBR Premix Ex Taq Kit (Toyobo, 4367659) on a QuantStudio 6 Flex (Applied Biosystems, USA) for 40 cycles according to the manufacturer’s protocol. *Gapdh* was used as an internal control. The data were analyzed with QuantStudio Real-Time PCR system software by the standard curve method. For the single AVNPC qPCR, the relative gene expression level was displayed with the heatmap of Ct values. The heatmap was generated using the “pheatmap” package (Version 1.0.12). Briefly, Ct values were scaled first, and the relative gene expression was reflected according to the colors in the scale bar. For the AVN tissues qPCR, the relative expression of mRNA level was quantified using 2^−ΔΔCT^ method. The primers used in qPCR assay are listed in Supplementary information, Table S2.

### ECG recordings in isolated hearts

ECG recordings were performed on isolated rat hearts (8–12 weeks old) as previously described.^46,47^ Briefly, the hearts were quickly removed from adult rats and hung on a modified Langendorff system with oxygenated perfusion solution containing 140 mM NaCl, 5.5 mM glucose, 5.4 mM KCl, 1.8 mM CaCl_2_, 1.2 mM K_2_HPO_4_, 5 mM HEPES and 1 mM MgCl_2_ (pH 7.4, adjusted with NaOH) at 37 °C through the aorta. For ECG recording, one electrode was placed at the base of the heart close to the right atrium, and another electrode was placed at the heart apex. The heart was paced through a pacing electrode (Powerlab, AD Instruments, USA) placed on the right atrium. The stimulation protocol was set as follows: the stimulation threshold was determined during the stabilization period and the pacing stimulus, typically 1.5–2 times the threshold, pulse duration of 2 ms and frequency of 6 Hz. The hearts were stabilized for 30 min before ECG recordings, and then different pharmacological drugs were added to the perfused solution. The ECG recordings were continuously obtained from Langendorff-perfused hearts with a Powerlab amplifier (Powerlab, AD Instruments, USA).

The AV block model was established by the addition of verapamil (250 nM) into the perfusion solution after 30 min equilibrium perfusion in isolated rat hearts. The AV block model was considered successful when isolated hearts developed second- or high-degree AV block that lasted more than 15 min.

### Telemetric ECG recordings

Telemetric ECG recordings were performed as previously described.^48,49^ Adult rats or mice (16–20 weeks old) were anesthetized with 2% isoflurane. Animals were implanted with an intraperitoneal telemetric ECG transmitter (Data Sciences International, St Paul, MN, USA) with paired wire electrodes placed over the thorax (lead II configuration). After surgery, the animals were housed in individual cages with free access to food and water. ECG signals were recorded using telemetry receivers for 24 h, and 7 days after recovery from surgical implantation, were analyzed using LabChart software (v8.1.9, ADInstruments Inc., CO, USA). ECG parameters, including P wave durations, PR intervals, QRS durations, and QT intervals were measured as previously described.^50^

### Optical mapping

Optical mapping was performed on isolated rat AVN tissues as described previously with modest modification.^51,52^ Briefly, rats were anesthetized with pentobarbital sodium (30 mg/kg). The rat heart was rapidly removed and placed in warm oxygenated Tyrode’s solution. Then, the AVN preparation, containing atrial and AVN tissue in the triangle of Koch, was dissected. The AVN preparation was stained with Di-4-ANEPPS (15 μM, AAT, 90134-00-2) for 40–60 min at 37 °C in oxygenated Tyrode’s solution. The blebbistatin (10 μM, MCE, HY-13813) was perfused for 10 min to eliminate motion artifacts during optical mapping recordings. Optical fluorescent signals were recorded at the triangle of Koch with a high-speed 10,000 pixels camera (SciMedia, MiCAM ULTIMA, USA) at 1000 frames/s under the right atrium pacing (5 Hz pacing rate, 2 times the threshold of pulse amplitude, 2 ms pulse duration). Before optical mapping assay, the AVN preparations were perfused with DMSO, Afloqualone (320 μM), Tiagabine (64 μM) or SKF89976A (128 μM) for 30–60 min. The AVN conduction time was calculated from the gradient of the activation maps.

### AAV2/9 vector construction

For AAV2/9 vectors, AAV2/9-CAG-MasterRNAi155 (*Gabrb2*)-mCherry-WPRE-pA (Taitool Biotech, AAV2/9-WY3556), AAV2/9-CAG-MasterRNAi155 (*Abat*)-mCherry-WPRE-pA (Taitool Biotech, AAV2/9-WY3583) and AAV2/9-CAG-MasterRNAi30e (*Slc32a1*)-mCherry-WPRE-pA (Taitool Biotech, AAV2/9-WY3603) were designed and constructed using standard methods by Taitool Biotech. The following oligonucleotide sequences were used for gene knockdown: *Gabrb2*-shRNA: 5′-GACAAAGATTGAGCTTCCTCA-3′; *Abat*-shRNA: 5′-ATGCATTCAAGACCATCTTCA-3′; *Slc32a1*-shRNA: 5′-TCCGGTTCCTAGTTGCTGATT-3′; scramble control for *Gabrb2*-shRNA and *Abat*-shRNA: 5′-GTCTCCACGCGCAGTACATTT-3′; scramble control for *Slc32a1*-shRNA: 5′-CGCTGAGTACTTCGAAATGTC-3′.

### Injection of AAV2/9 vectors into AVN tissue

To effectively infect AVN with AAV2/9 virus, the virus was injected into AVN tissue directly as described previously.^53^ In brief, adult rats (8–12 weeks old) were anesthetized with pentobarbital sodium (30 mg/kg) by intraperitoneal injection, and then the hair on the thorax was shaved. The rats were incubated with cannulas connected to a rodent ventilator. Then, the heart was exposed via thoracotomy at the fourth right intercostal space. A fat pad with a diameter of 5 mm on the epicardial surface of the right atrium wall and the aortic root was identified under the stereomicroscope. The insertion point was 1 mm posterolateral to the fat pad. During virus injection, the needle of the insulin syringe was bent at a 90° angle and at 3 mm from the tip of the needle, then the insulin syringe was inserted parallel to the aorta toward the apex until reaching the AVN region. A transient AV block could be recorded by ECG when the needle of the insulin syringe was injected into the AVN region, and a dose of 8 × 10^11^ viral particles was administered. After injection, the chest was closed with 4.0 silk suture, and the skin was then closed with interrupted sutures. The rat was disconnected from the rodent ventilator, and then kept on a heating pad until recovery of consciousness. After 8 weeks, telemetric ECG recordings were performed in rats injected with AAV2/9 virus.

### Intracardiac electrophysiological recordings in vivo

Intracardiac electrophysiological recordings in vivo were performed as previously described.^6,54^ For surface ECG examinations, mice were anesthetized with 2% isoflurane. The needles were inserted subcutaneously to the limbs and connected with electrodes to record ECG signals using LabChart software and Bio Amplifier (AD Instruments, Colorado Springs, CO, USA). For intracardiac ECG recordings and stimulation, an octopolar electrode catheter (EPA-800, Millar, Houston, TX, USA) was applied through the right jugular vein, advanced into the right atrium and subsequently into the right ventricle. The position of electrode was identified by the morphology of the intracardiac ECGs. Standard clinical protocols were used to determine AVN conduction properties. To evaluate the atrioventricular WBP and 2:1 AV conduction, a train of 8 atrial stimuli was repeated while the PCL was reduced from 90 ms to 40 ms by 2 ms steps. WBP was identified by the longest S1S1 coupling interval at which AV block was observed. The 2:1 AV conduction was identified as the longest S1S1 interval at which 2:1 AV block was observed. To measure the AVNERP, a train of 8 atrial stimuli at a certain PCL (S1S1 = 100, 90, and 80 ms) was followed by one extrastimulus S2. The S1S1 coupling interval was reduced from 70 ms to 20 ms by 2 ms steps. AVNERP was defined as the longest S1S2 coupling interval with loss of ventricular signals. To determine whether Afloqualone could rescue the alterations of AVN conduction properties in *Gabrb2*-CKO mice, intracardiac administration of Afloqualone (320 μM) was applied. The ventricular fatal arrhythmias (VT/VF) were conducted using the atrial burst pacing at 50 Hz 20 times after the subcutaneous injection of 2.5 mg/kg isoproterenol (Sigma, I5627). Ventricular fatal arrhythmia was identified as a rapid, irregular ventricular rhythm.

### Intracardiac electrophysiological recordings ex vivo

Intracardiac electrophysiological recordings ex vivo were performed as previously described.^6^ Briefly, the hearts were dissected, cannulated via the aorta and retrograde perfused with 37 °C Tyrode’s solution containing 140 mM NaCl, 5.5 mM glucose, 5.4 mM KCl, 1.8 mM CaCl_2_, 1.2 mM K_2_HPO_4_, 5 mM HEPES and 1 mM MgCl_2_ (pH 7.4, adjusted with NaOH). For ECG recordings, one electrode was placed at the base of the heart close to the right atrium, and another electrode was placed at the heart apex connected with LabChart software and Bio Amplifier (AD Instruments, Colorado Springs, CO, USA). The heart was paced by an octopolar electrode catheter (EPA-800, Millar, Houston, TX, USA) at the junction between the superior vena cava and right atrium. The Standard clinical protocols were used to determine the AVN conduction properties as carried out in intracardiac electrophysiological recordings. WBP and 2:1 AV conduction were determined by a train of 8 atrial stimuli while the PCL was reduced from 120 ms to 40 ms by 2 ms steps. AVNERP was determined by a train of 8 atrial stimuli (S1S1 = 120) followed by one extrastimulus S2. The S1S2 coupling interval was reduced from 100 ms to 40 ms by 2 ms steps.

### Echocardiography

Transthoracic echocardiography was performed with the Vevo 2100 High-Resolution. Micro Imaging System was performed on mice under 1% isoflurane anesthesia in vivo. Cardiac systolic functions including EF% and FS% were calculated from the parasternal long-axis M-mode view.

### Immunofluorescence

For immunocytochemistry, the isolated AVNPCs were fixed in 4% paraformaldehyde (PFA) for 15 min and washed with PBS twice. Then the cells were permeabilized with 0.5% Triton X-100 in PBS for 10 min, washed in PBS twice and blocked with 4% goat serum for 1 h at room temperature. For immunostaining, cells were incubated with primary antibodies overnight at 4 °C. On the second day, after washing with PBST twice, the cells were incubated with suitable secondary antibodies for 1 h at room temperature, followed by 30 min of 4,6-diamidino-2-phenylindole dihydrochloride (DAPI) staining. Representative images were taken using a Leica confocal microscope.

For immunohistochemistry, the hearts of adult mice or rats were quickly excised, fixed with 4% PFA overnight at 4 °C and embedded in paraffin. The hearts were then sectioned longitudinally in 6 μm slices for immunostaining. The heart sections underwent deparaffinization with xylene and then rehydrated in decreasing concentrations of ethanol, followed by antigen retrieval in citric acid buffer. The sections were blocked with 5% goat serum for 1 h at room temperature, and then were incubated at 4 °C overnight with primary antibodies diluted in 5% goat serum. After three washes in 0.1% PBST, the sections were stained for 1 h at room temperature with fluorescent secondary antibodies followed by 10 min DAPI staining for nuclear visualization. Representative images were taken using a Leica confocal microscope.

The following primary antibodies were used in immunofluorescence experiments: anti-GABA antibody (5A9) (Abcam, ab86186, 1:100 for cells); anti-GABRA3 antibody (Alomone, AGA-003, 1:200 for cells, 1:50 for heart slices); anti-GABRB2 antibody (Invitrogen, PA5-64331, 1:100 for cells, 1:20 for heart slices); anti-GABRG2 antibody (Alomone, AGA-005, 1:100 for cells, 1:50 for heart slices); anti-GAT-1 antibody (Synaptic Systems, 274102, 1:200 for cells, 1:50 for heart slices); anti-vGAT antibody (Alomone, AGT-005, 1:100 for cells, 1:50 for heart slices); anti-GAD2 antibody (Santa Cruz Biotechnology, sc-377145, 1:100 for cells, 1:50 for heart slices); anti-GABA-T antibody (Abcam, ab216465, 1:100 for cells, 1:50 for heart slices); anti-SSADH antibody (Santa Cruz Biotechnology, sc-390754, 1:100 for cells, 1:50 for heart slices); anti-CX43 antibody (Cell Signaling Technology, 3512, 1:50 for heart slices); anti-HCN4 antibody produced in mouse (Sigma, SAB5200035, 1:100 for cells, 1:50 for heart slices); anti-HCN4 antibody produced in rat (Sigma, MABN1871, 1:100 for cells, 1:50 for heart slices); and anti-CAST antibody (Invitrogen, PA5-87352, 1:100 for cells, 1:50 for heart slices).

### Electron microscopy

Fresh heart tissues (< 1 mm^3^) were fixed in 5 mL fixation buffer (2.5% glutaraldehyde, 2.0% PFA in 0.1 M sodium phosphate buffer, pH 7.4) overnight at 4 °C. Samples were processed as previously described before examination with an electron microscope under 80 kV. Images were acquired with TEM (JOEL TEM1230, Japan).

### Statistical analysis

Statistical analysis was performed with GraphPad Prism 9 statistical software. All statistical data are shown as means ± SD. Two-tailed unpaired Student’s *t*-test was used for the statistical analysis of two groups. One-way ANOVA followed by Dunnett’s multiple comparison test was used for the statistical analysis of more than two groups. Significant differences in the proportion of second- or high-degree AV block occurrence between the AAV2/9-*Control-*, AAV2/9-*Gabrb2-*, and AAV2/9-*Slc32a1*-injected groups were determined using the Fisher’s exact test. For the concentration-response curves and IC_50_/EC_50_ evaluations, the log (inhibitor/agonist) vs normalized response–variable slope model was used. The *P* < 0.05 was considered statistically significant. The exact *P* values are shown in the corresponding figures.

### Supplementary information

Supplementary information, Fig. S1

Supplementary information, Fig. S2

Supplementary information, Fig. S3

Supplementary information, Fig. S4

Supplementary information, Fig. S5

Supplementary information, Fig. S6

Supplementary information, Fig. S7

Supplementary information, Fig. S8

Supplementary information, Fig. S9

Supplementary information, Fig. S10

Supplementary information, Fig. S11

Supplementary information, Fig. S12

Supplementary information, Fig. S13

Supplementary information, Fig. S14

Supplementary information, Fig. S15

Supplementary information, Fig. S16

Supplementary information, Fig. S17

Supplementary information, Fig. S18

Supplementary information, Fig. S19

Supplementary information, Table S1

Supplementary information, Table S2
