# Supplementary material for: A GABAergic system in atrioventricular node pacemaker cells controls electrical conduction between the atria and ventricles
Source: Cell Res. 2024 Jun 7;34(8):556–71. doi: 10.1038/s41422-024-00980-x (PMC11291642; doi:10.1038/s41422-024-00980-x)
Supplement: Supplementary file 1 — Supplementary information, Fig. S1 [file 41422_2024_980_MOESM1_ESM.pdf]

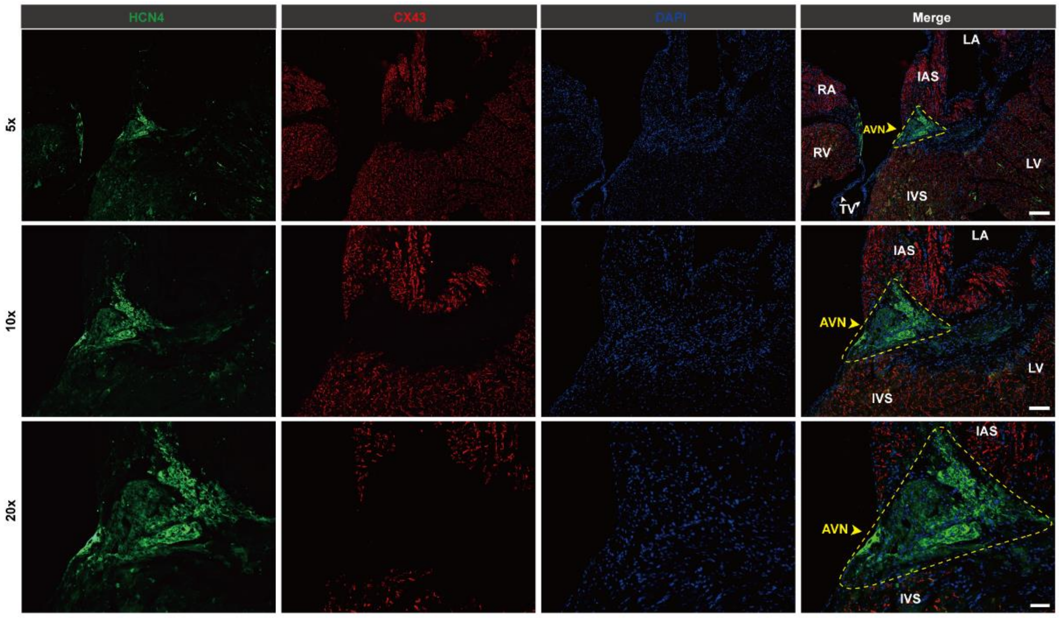

Supplementary information, Fig. S1 The identification of atrioventricular node. Immunofluorescence staining showing the location of the atrioventricular node (AVN) in adult mouse heart. HCN4, green; CX43, red; DAPI, blue. For 5x magnification images, Scale bar, 200 µm; for 10x magnification images, Scale bar, 100 µm; for 20x magnification images, Scale bar, 50 µm. LA, left atrium; LV, left ventricle; RA, right atrium; RV, right ventricle; IAS, interatrial septum; IVS, interventricular septum; TV, tricuspid valve; AVN, atrioventricular node.
