# Supplementary material for: A GABAergic system in atrioventricular node pacemaker cells controls electrical conduction between the atria and ventricles
Source: Cell Res. 2024 Jun 7;34(8):556–71. doi: 10.1038/s41422-024-00980-x (PMC11291642; doi:10.1038/s41422-024-00980-x)
Supplement: Supplementary file 2 — Supplementary information, Fig. S2 [file 41422_2024_980_MOESM2_ESM.pdf]

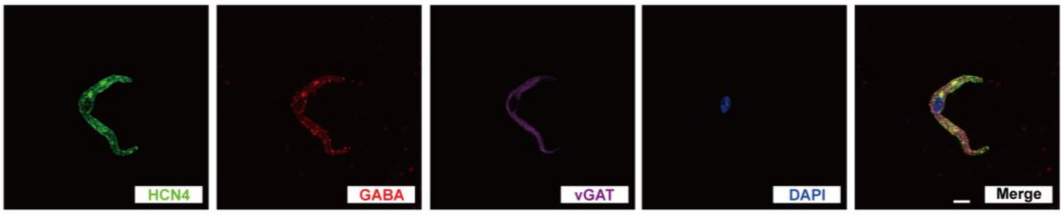

Supplementary information, Fig. S2 Immunofluorescence images showing the colocalization of GABA with vesicular GABA transporter (vGAT) in single rat atrioventricular node pacemaker cell. Scale bar, 10 μm.
