# Supplementary material for: A GABAergic system in atrioventricular node pacemaker cells controls electrical conduction between the atria and ventricles
Source: Cell Res. 2024 Jun 7;34(8):556–71. doi: 10.1038/s41422-024-00980-x (PMC11291642; doi:10.1038/s41422-024-00980-x)
Supplement: Supplementary file 3 — Supplementary information, Fig. S3 [file 41422_2024_980_MOESM3_ESM.pdf]

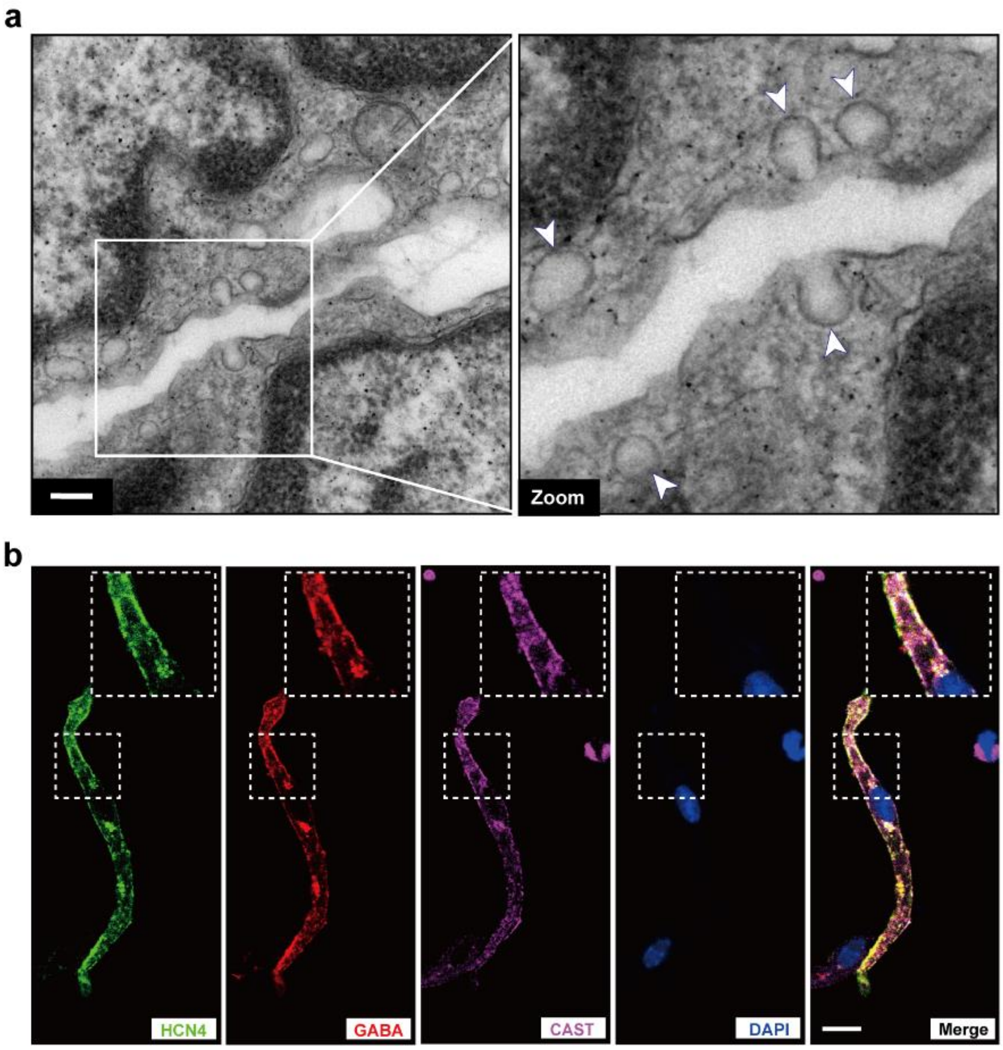

Supplementary information, Fig. S3 GABA-containing vesicles exist in mouse atrioventricular node pacemaker cell.

a Transmission electron microscopy (TEM) examination of adult mice atrioventricular node (AVN) tissue. Representative electron microscopy images showing transmitter vesicle-like ultrastructures beneath the plasma membrane of mouse atrioventricular node pacemaker cells (AVNPCs). Right, a magnified version of the white box in the left image. White arrows indicate the vesicles. Scale bar, 200 nm. **b** Immunofluorescence images showing the colocalization of GABA with the vesicle marker CAST in mouse AVNPC. Scale bar, 10 μm.
