# Supplementary material for: A GABAergic system in atrioventricular node pacemaker cells controls electrical conduction between the atria and ventricles
Source: Cell Res. 2024 Jun 7;34(8):556–71. doi: 10.1038/s41422-024-00980-x (PMC11291642; doi:10.1038/s41422-024-00980-x)
Supplement: Supplementary file 4 — Supplementary information, Fig. S4 [file 41422_2024_980_MOESM4_ESM.pdf]

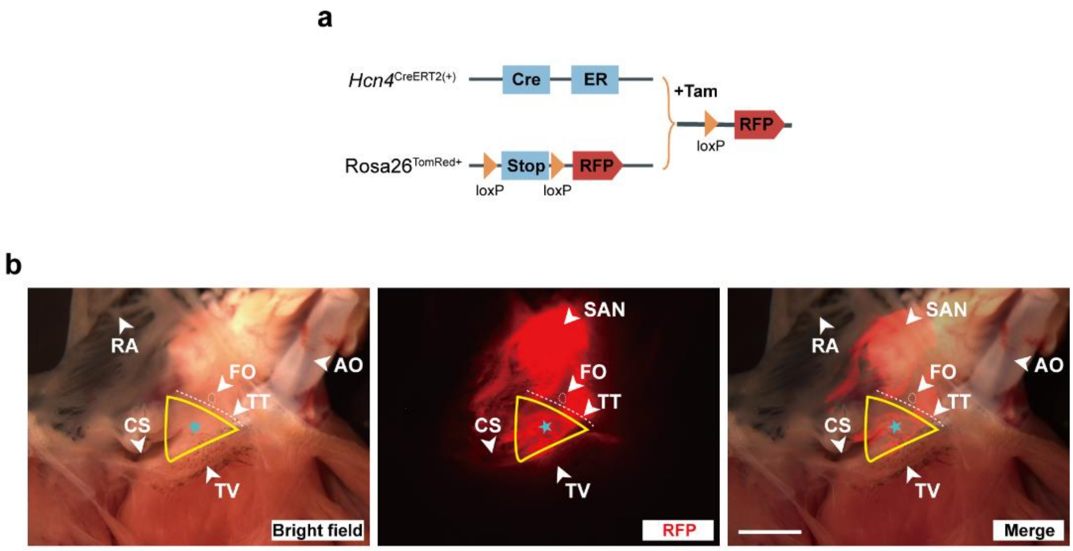

Supplementary information, Fig. S4 Strategy for the generation of *Hcn4*$^{\mathrm{CreERT2(+)}}$; Rosa26$^{\mathrm{TomRed+}}$ mice.

a Schematic diagram showing the generation of the *Hcn4*$^{\mathrm{CreERT2(+)}}$; Rosa26$^{\mathrm{TomRed+}}$ mouse line. Cre recombination was used to induce the expression of TomRed in the cardiac conduction system of *Hcn4*$^{\mathrm{CreERT2(+)}}$; Rosa26$^{\mathrm{TomRed+}}$mice. Tam, Tamoxifen. **b** Representative images of atrioventricular node (AVN) in adult *Hcn4*$^{\mathrm{CreERT2(+)}}$; Rosa26$^{\mathrm{TomRed+}}$mice at 2 weeks after tamoxifen administration. Brightfield (*left*), TomRed fluorescence (*middle*) and merged image (*right*) of the *Hcn4*$^{\mathrm{CreERT2(+)}}$; Rosa26$^{\mathrm{TomRed+}}$ heart indicate the TomRed expressionin the AVN region. Yellow triangle indicates the triangle of Koch area. Asterisk denotes the AVN location. Scale bar, 2 mm. FO, fossa ovalis; TV, tricuspid valve; TT, tendon of Todaro; CS, coronary sinus; AO, aorta; RA, right atrium; SAN, sinoatrial node.
