# Supplementary material for: A GABAergic system in atrioventricular node pacemaker cells controls electrical conduction between the atria and ventricles
Source: Cell Res. 2024 Jun 7;34(8):556–71. doi: 10.1038/s41422-024-00980-x (PMC11291642; doi:10.1038/s41422-024-00980-x)
Supplement: Supplementary file 6 — Supplementary information, Fig. S6 [file 41422_2024_980_MOESM6_ESM.pdf]

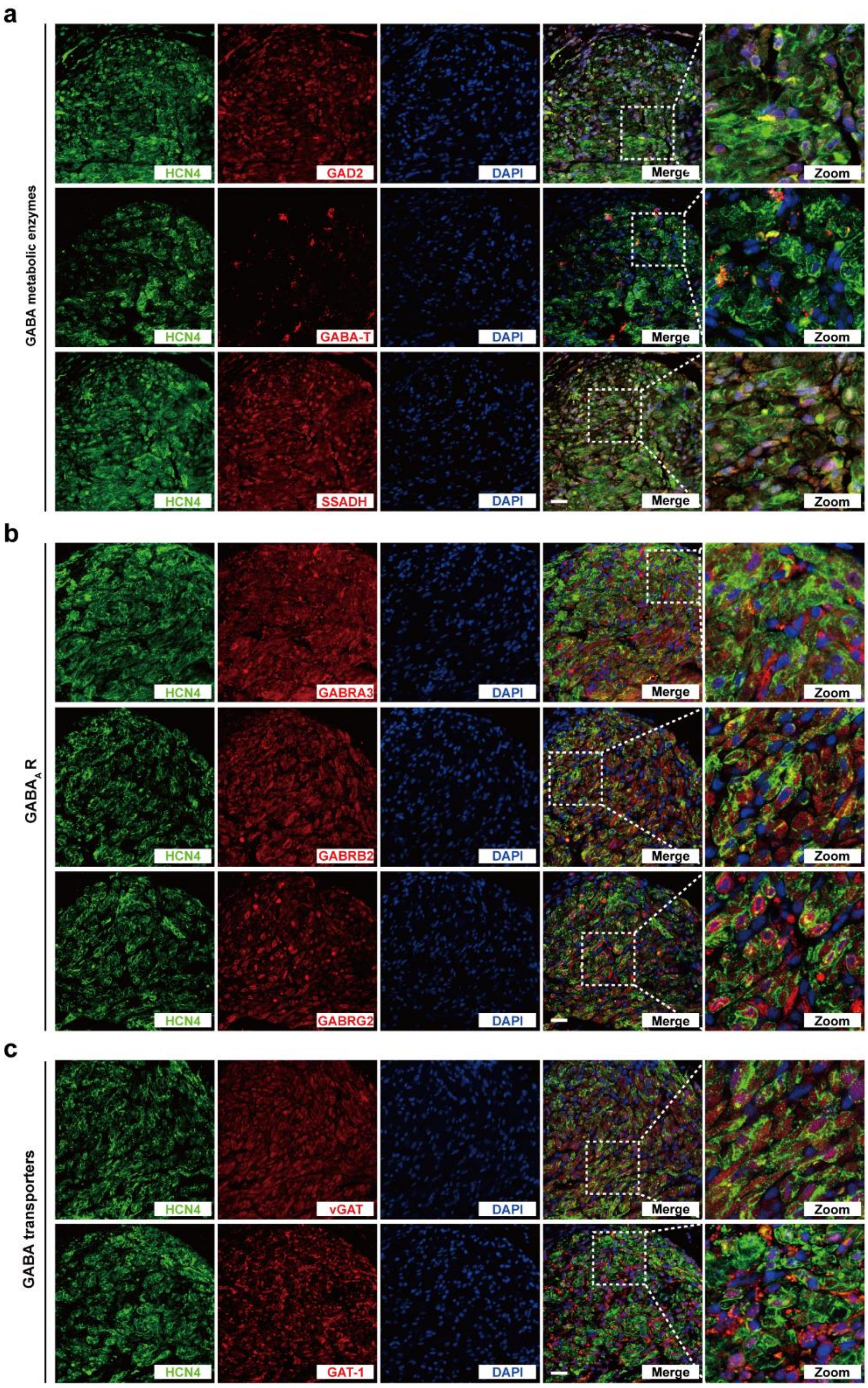

Supplementary information, Fig. S6 The expression of key elements of the

GABAergic system in rat atrioventricular node tissue.

a Immunofluorescence staining of GABA metabolic enzymes (GAD2, GABA-T and SSADH) in rat atrioventricular node (AVN) tissue. Scale bar, 25 μm. **b** Immunofluorescent staining of $GABA_A$ receptor (GABRA3, GABRB2 and GABRG2) in rat AVN tissue. Scale bar, 25 μm. **c** Immunofluorescence staining of GABA transporters (vGAT and GAT-1) in rat AVN tissue. $GABA_AR$, $GABA_A$ receptor. Scale bar, 25 μm.
