# Supplementary material for: A GABAergic system in atrioventricular node pacemaker cells controls electrical conduction between the atria and ventricles
Source: Cell Res. 2024 Jun 7;34(8):556–71. doi: 10.1038/s41422-024-00980-x (PMC11291642; doi:10.1038/s41422-024-00980-x)
Supplement: Supplementary file 7 — Supplementary information, Fig. S7 [file 41422_2024_980_MOESM7_ESM.pdf]

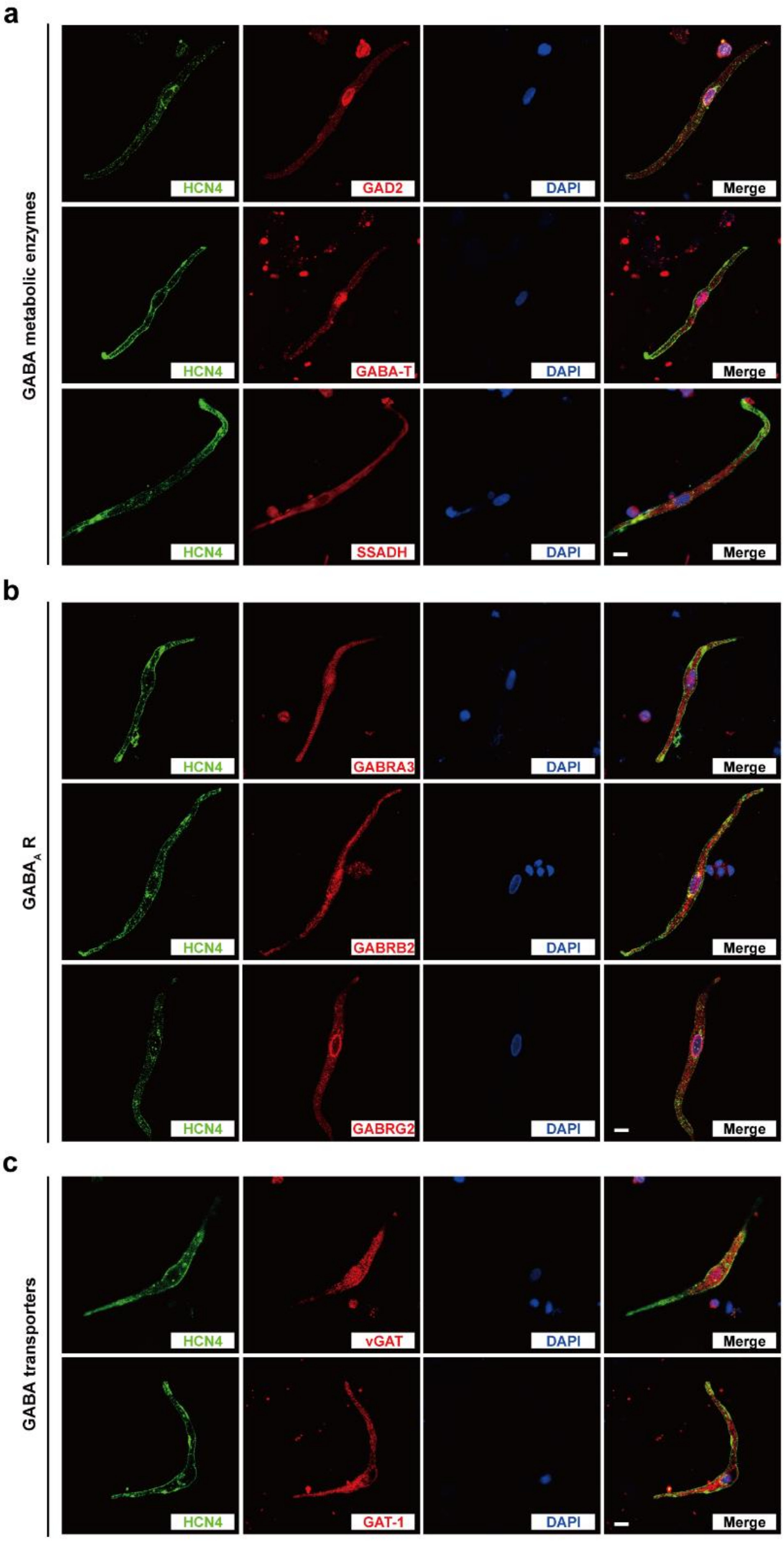
a
GABA metabolic enzymes
HCN4
GAD2
DAPI
Merge
HCN4
GABA-T
DAPI
Merge
HCN4
SSADH
DAPI
Merge
b
GABA$_A$ R
HCN4
GABRA3
DAPI
Merge
HCN4
GABRB2
DAPI
Merge
HCN4
GABRG2
DAPI
Merge
c
GABA transporters
HCN4
vGAT
DAPI
Merge
HCN4
GAT-1
DAPI
Merge

Supplementary information, Fig. S7 The expression of key elements of the GABAergic system in rat atrioventricular node pacemaker cells.

a Immunofluorescence staining showing the expression and localization of GABA metabolic enzymes (GAD2, GABA-T and SSADH) in rat single atrioventricular node pacemaker cells (AVNPCs). Scale bar, 10 μm. **b** Immunofluorescence staining showing the expression and localization of $GABA_A$ receptor (GABRA3, GABRB2 and GABRG2) in rat AVNPCs. Scale bar, 10 μm. **c** Immunofluorescence staining showing the expression and localization of GABA transporters (vGAT and GAT-1) in rat single AVNPCs. $GABA_AR$, $GABA_A$ receptor. Scale bar, 10 μm.
