# Supplementary material for: A GABAergic system in atrioventricular node pacemaker cells controls electrical conduction between the atria and ventricles
Source: Cell Res. 2024 Jun 7;34(8):556–71. doi: 10.1038/s41422-024-00980-x (PMC11291642; doi:10.1038/s41422-024-00980-x)
Supplement: Supplementary file 8 — Supplementary information, Fig. S8 [file 41422_2024_980_MOESM8_ESM.pdf]

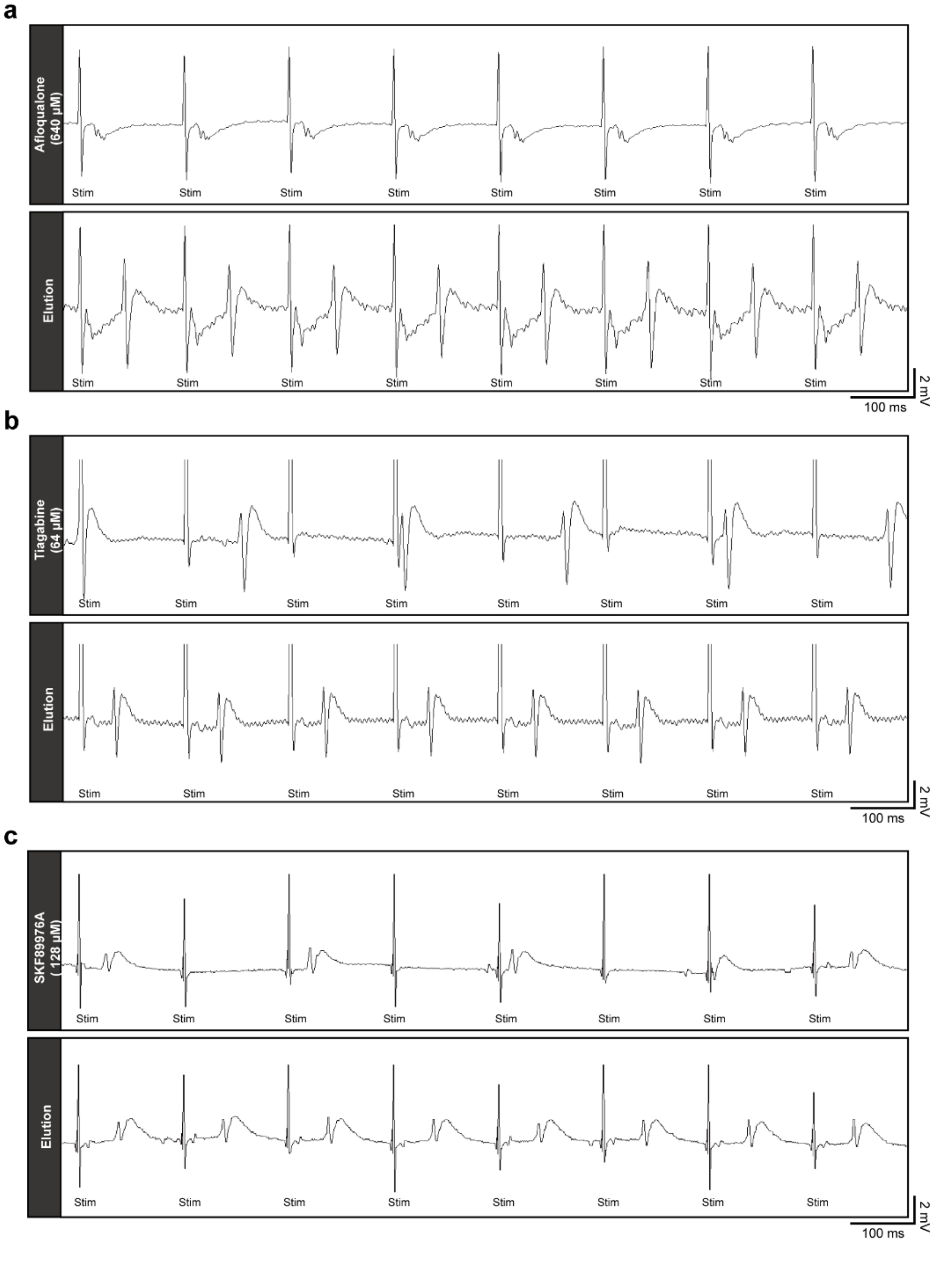


**Supplementary information, Fig. S8 The intervention targeting the GABAergic system leads to severe atrioventricular block in isolated rat hearts.**

**a** Representative electrocardiogram (ECG) recording showing the hearts suffering cardiac arrest when perfused with the $GABA_A$ receptor agonist Afloqualone at 640 μM (*top*) and returning to normal ECGs after stopping the Afloqualone perfusion *(bottom*).

Scale bars are 100 ms horizontal and 2 mV vertical. Stim, stimulation. **b** Representative ECG recordings showing that a high concentration of the GABA reuptake inhibitor Tiagabine (64 μM) could cause third-degree atrioventricular block (AV block) in the perfused hearts (*top*), and normal PR intervals could be restored after Tiagabine perfusion is stopped (*bottom*). Scale bars are 100 ms horizontal and 2 mV vertical. Stim, stimulation. **c** Representative ECG recordings showing that a high concentration of the GABA transporter-1 inhibitor SKF89976A (128 μM) could cause third-degree AV block in the perfused hearts (*top*), and normal PR intervals could be restored after stopping SKF89976A perfusion (*bottom*). Scale bars are 100 ms horizontal and 2 mV vertical. Stim, stimulation.
