# Supplementary material for: A GABAergic system in atrioventricular node pacemaker cells controls electrical conduction between the atria and ventricles
Source: Cell Res. 2024 Jun 7;34(8):556–71. doi: 10.1038/s41422-024-00980-x (PMC11291642; doi:10.1038/s41422-024-00980-x)
Supplement: Supplementary file 9 — Supplementary information, Fig. S9 [file 41422_2024_980_MOESM9_ESM.pdf]

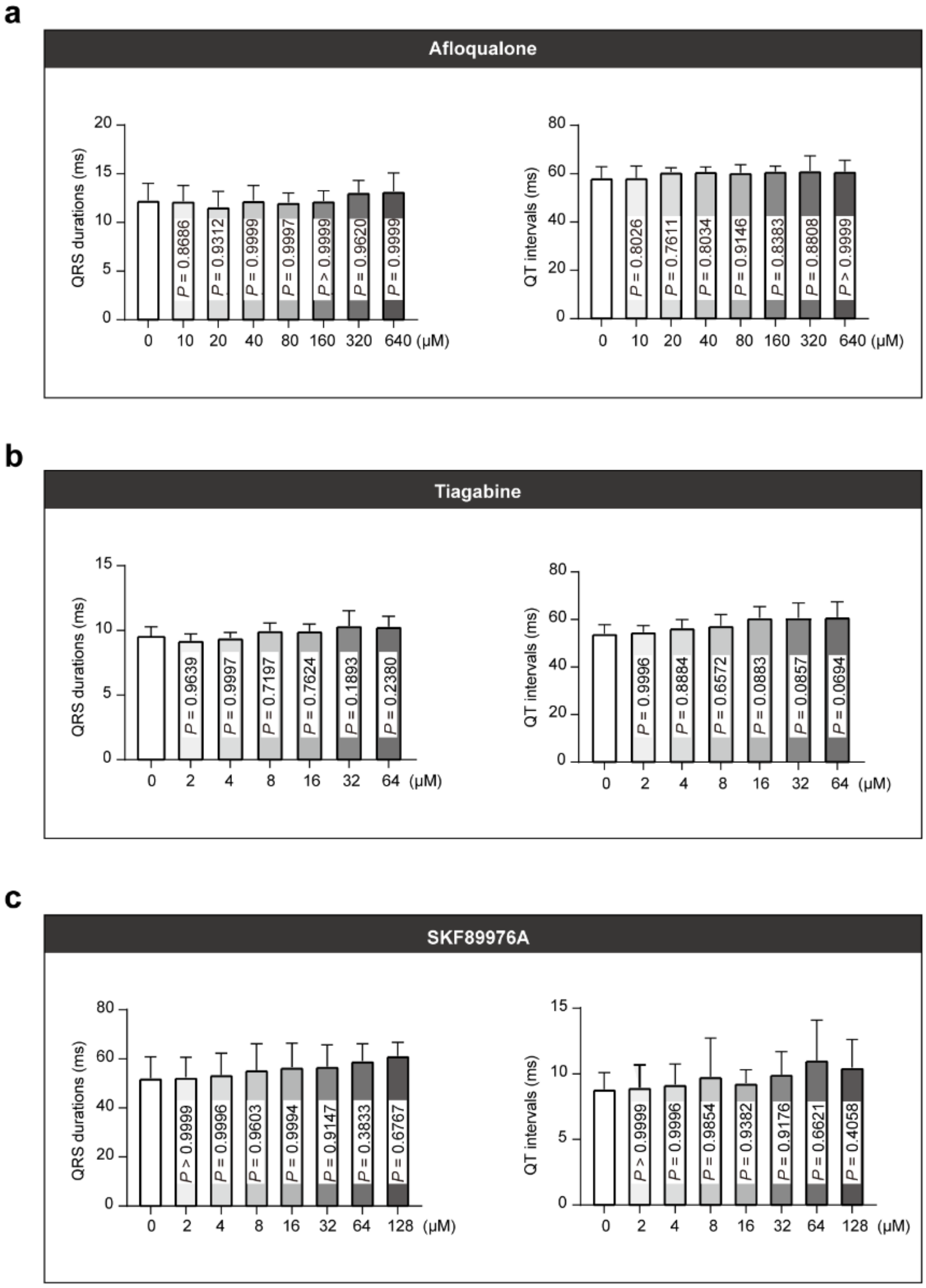

Supplementary information, Fig. S9 The QRS durations and QT intervals are not changed after the administration of GABAergic system agonist or inhibitors in isolated rat hearts.

a-c Columns showing the quantification of QRS durations and QT intervals in perfused rat hearts treated with different concentrations of $GABA_A$ receptor agonist

(Afloqualone) (**a**), GABA reuptake inhibitor (Tiagabine) (**b**) and GABA transporter-1 inhibitor (SKF89976A) (**c**) under right atrial pacing (6 Hz). n = 5 hearts for Afloqualone and SKF89976A treatment group, n = 6 hearts for Tiagabine treatment group. Data are presented as the mean ± s.d.. P values were calculated by one-way ANOVA with Dunnett's multiple comparisons test.
