# Supplementary material for: A GABAergic system in atrioventricular node pacemaker cells controls electrical conduction between the atria and ventricles
Source: Cell Res. 2024 Jun 7;34(8):556–71. doi: 10.1038/s41422-024-00980-x (PMC11291642; doi:10.1038/s41422-024-00980-x)
Supplement: Supplementary file 10 — Supplementary information, Fig. S10 [file 41422_2024_980_MOESM10_ESM.pdf]

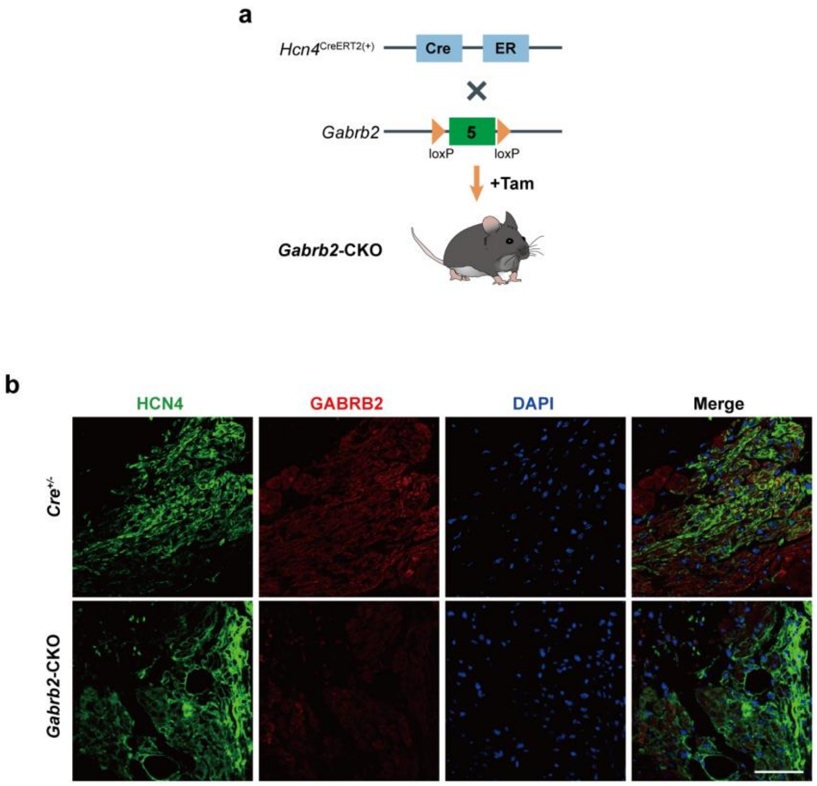

Supplementary information, Fig. S10 The generation of the cardiac conduction system-specific *Gabrb2* knockout (*Gabrb2*-CKO) mouse line.

a Schematic of the generation of mouse line harbouring cardiac conduction system-specific knockdown (CKO) of *Gabrb2* gene using the *Hcn4*$^{\mathrm{CreERT2(+)}}$ mouse line. Tam, Tamoxifen. **b** Immunofluorescence staining showing the effective knockout of *Gabrb2* in the AVN of *Gabrb2*-CKO mice compared with that in *Cre*$^{+/-}$ mice. Scale bar, 50 μm.
