# Supplementary material for: A GABAergic system in atrioventricular node pacemaker cells controls electrical conduction between the atria and ventricles
Source: Cell Res. 2024 Jun 7;34(8):556–71. doi: 10.1038/s41422-024-00980-x (PMC11291642; doi:10.1038/s41422-024-00980-x)
Supplement: Supplementary file 11 — Supplementary information, Fig. S11 [file 41422_2024_980_MOESM11_ESM.pdf]

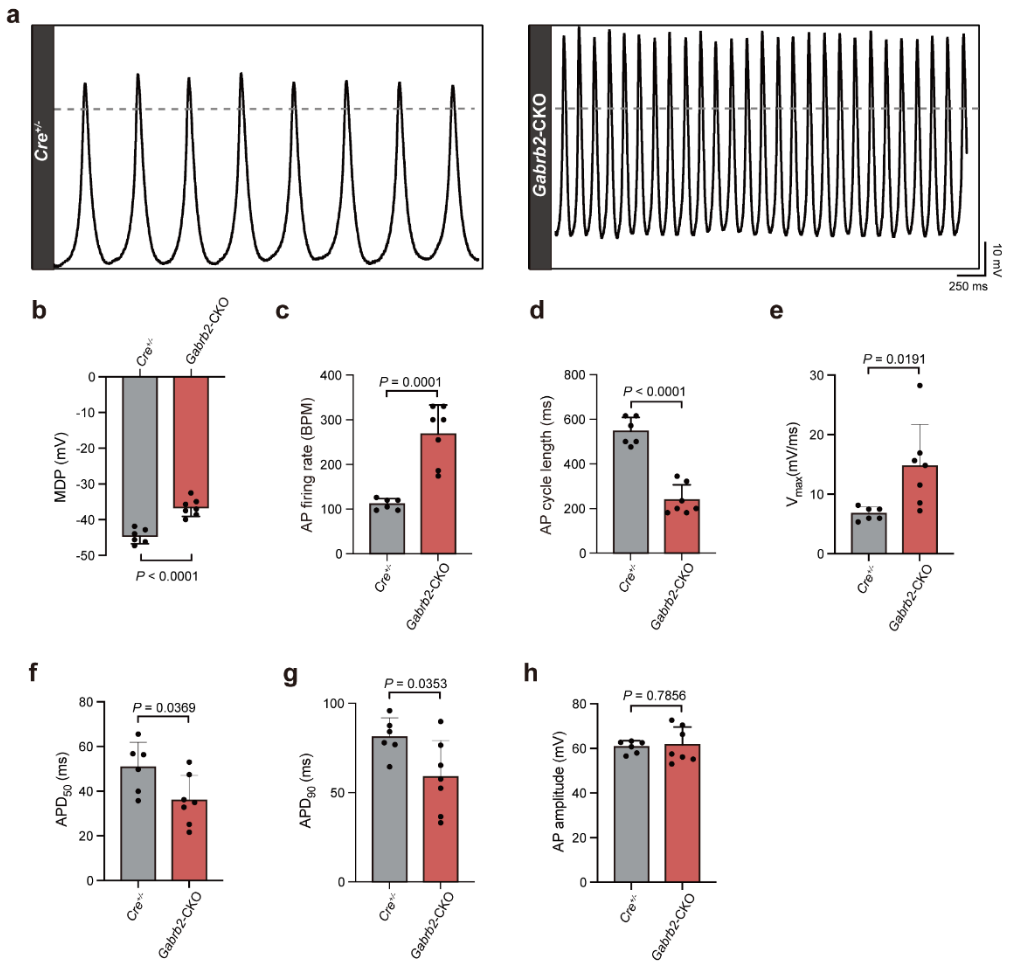

Supplementary information, Fig. S11 *Gabrb2* knockout increase the excitability of atrioventricular node pacemaker cells.

a Representative spontaneous action potentials (APs) recorded in *Cre*$^{+/-}$ and *Gabrb2*-CKO mice atrioventricular node pacemaker cells (AVNPCs) using current patch-clamp. The horizontal line marks 0 mV. Scale bars are 250 ms horizontal and 10 mV vertical. **b-h** Pooled data of maximum diastolic potential (MDP), spontaneous AP firing rate, AP cycle length, max upstroke velocity (V_{max}), AP duration measure at 50% (APD_{50}) and 90% (APD_{90}) repolarization, and AP amplitude from AP recording using current patch clamp in *Cre*$^{+/-}$ and *Gabrb2*-CKO mice AVNPCs. Data are shown as the mean ± s.d.. *P*

values were calculated by two-tailed unpaired student t test. *n* = 6 cells for *Cre*$^{+/-}$ mice and *n* = 7 cells for *Gabrb2*-CKO mice.
