# Supplementary material for: A GABAergic system in atrioventricular node pacemaker cells controls electrical conduction between the atria and ventricles
Source: Cell Res. 2024 Jun 7;34(8):556–71. doi: 10.1038/s41422-024-00980-x (PMC11291642; doi:10.1038/s41422-024-00980-x)
Supplement: Supplementary file 12 — Supplementary information, Fig. S12 [file 41422_2024_980_MOESM12_ESM.pdf]

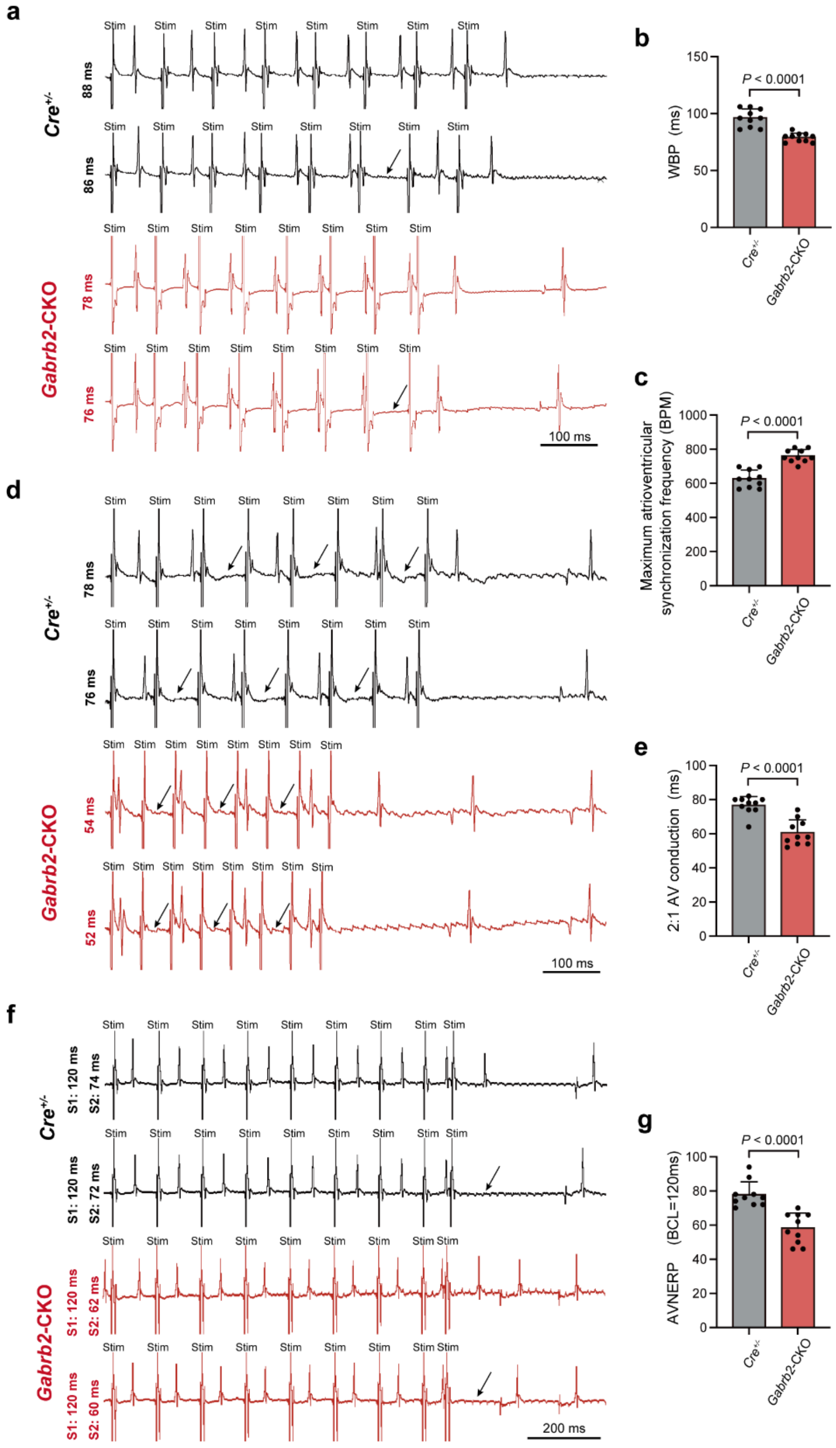

Supplementary information, Fig. S12 *Gabrb2* knockout accelerates the electrical conduction between the atria and the ventricles *ex vivo*.

a-e Intracardiac programmed electrical stimulation (PES) was used to examine the electrical physiological function parameters of AVN including Wenckebach periodicity

(WBP) (**a, b**), maximum atrioventricular synchronization frequency (**c**) and 2:1 atrioventricular conduction (2:1 AV conduction) (**d, e**) from *Cre*$^{+/-}$ and *Gabrb2*-CKO mice *ex vivo*. **a, d** Representative ECG traces for evaluation of the above parameters by PES. The drop of ventricular QRS complex was pointed by the arrows. The 2:1 AV conduction indicates the Wenckebach point that only one QRS complex was generated by two S1 stimulations. **f, g** Representative images and quantification of atrioventricular node effective refractory period (AVNERP). Arrows indicate loss of S2 induced ventricular signal. Data are shown as mean ± s.d.. *P* values were calculated using two-tailed unpaired student t test. *n* = 10 mice per group. Stim, stimulation.
