# Supplementary material for: A GABAergic system in atrioventricular node pacemaker cells controls electrical conduction between the atria and ventricles
Source: Cell Res. 2024 Jun 7;34(8):556–71. doi: 10.1038/s41422-024-00980-x (PMC11291642; doi:10.1038/s41422-024-00980-x)
Supplement: Supplementary file 13 — Supplementary information, Fig. S13 [file 41422_2024_980_MOESM13_ESM.pdf]

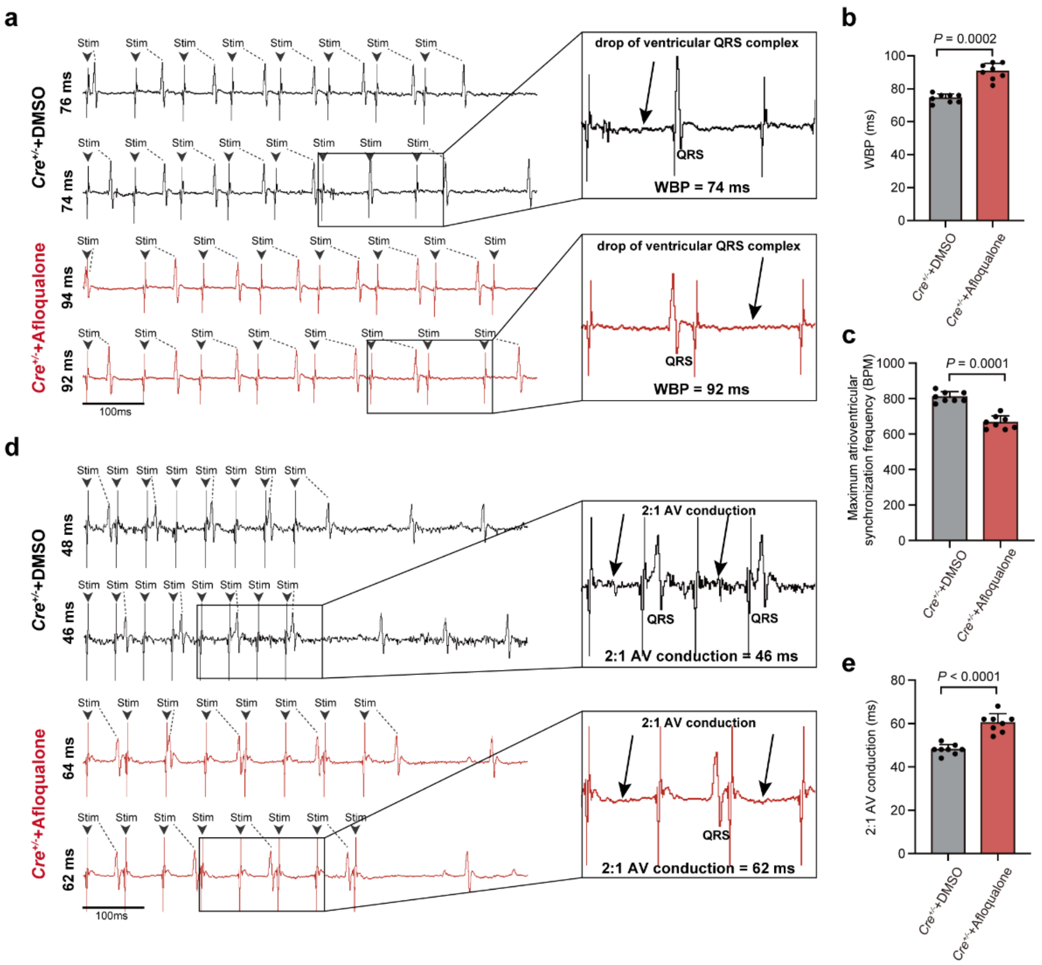

Supplementary information, Fig. S13 Afloqualone decelerates the electrical conduction between the atria and the ventricles. a-e Intracardiac programmed electrical stimulation (PES) was used to examine the electrical physiological function parameters of AVN including Wenckebach periodicity (WBP) (**a, b**), maximum atrioventricular synchronization frequency (**c**) and 2:1 atrioventricular conduction (2:1 AV conduction) (**d, e**) from DMSO-treated and Afloqualone-treated *Cre*$^{+/-}$ mice. DMSO, control solvent group. **a, d** Representative ECG traces for evaluation of the above parameters by PES. The drop of ventricular QRS complex pointed by the arrows is highlighted in the black box. The 2:1 AV conduction indicates the Wenckebach point that only one QRS complex was generated by two S1 stimulations. Data are shown as

mean ± s.d.. *P* values were calculated using two-tailed unpaired student t test. *n* = 8 mice per group. Stim, stimulation.
