# Supplementary material for: A GABAergic system in atrioventricular node pacemaker cells controls electrical conduction between the atria and ventricles
Source: Cell Res. 2024 Jun 7;34(8):556–71. doi: 10.1038/s41422-024-00980-x (PMC11291642; doi:10.1038/s41422-024-00980-x)
Supplement: Supplementary file 16 — Supplementary information, Fig. S16 [file 41422_2024_980_MOESM16_ESM.pdf]

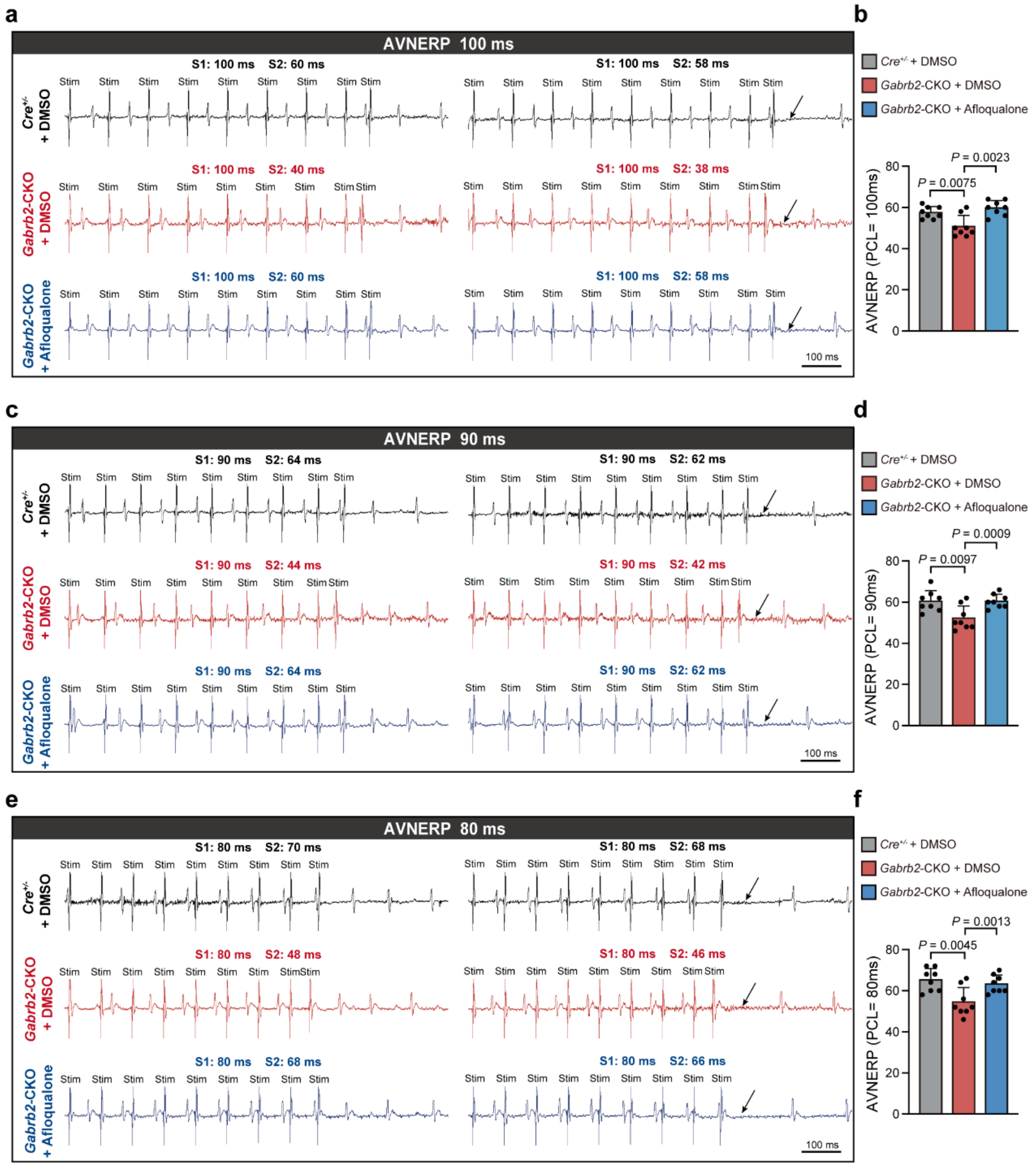

Supplementary information, Fig. S16 Afloqualone attenuates the effect of *Gabrb2* deficiency on atrioventricular conduction evaluated by atrioventricular node effective refractory period.

a-f The atrioventricular node effective refractory period (AVNERP) was evaluated by programmed electrical stimulus (PES) under different pacing cycle length (PCL) in *Cre*$^{+/-}$ + DMSO, *Gabrb2*-CKO + DMSO and *Gabrb2*-CKO + Afloqualone mice. The

short trains of 8 atrial stimulus (S1) followed by 1 extra stimulus (S2) were applied. DMSO, control solvent group. Arrows indicate loss of S2 induced ventricular signal. Data are shown as mean ± s.d.. *P* values were calculated using one-way ANOVA with Dunnett's multiple comparisons test. *n* = 8 mice per group. Stim, stimulation.
