# Supplementary material for: A GABAergic system in atrioventricular node pacemaker cells controls electrical conduction between the atria and ventricles
Source: Cell Res. 2024 Jun 7;34(8):556–71. doi: 10.1038/s41422-024-00980-x (PMC11291642; doi:10.1038/s41422-024-00980-x)
Supplement: Supplementary file 17 — Supplementary information, Fig. S17 [file 41422_2024_980_MOESM17_ESM.pdf]

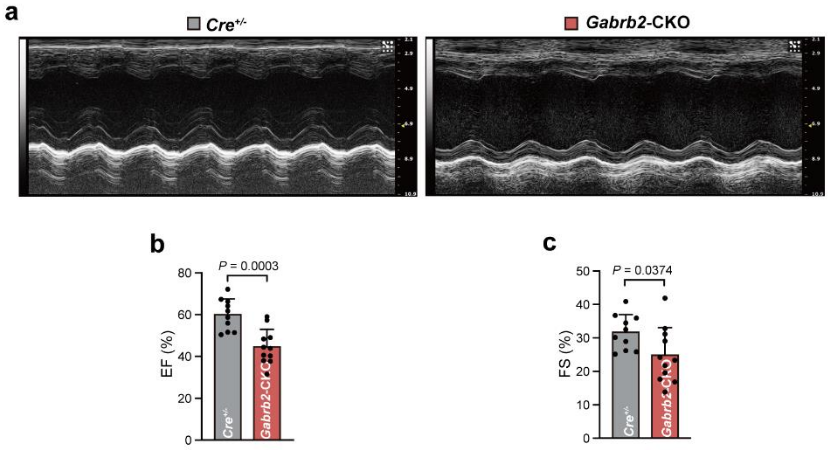

Supplementary information, Fig. S17 *Gabrb2* knockout results in impaired ventricular contractile function. a Representative M-mode echocardiogram of *Cre*$^{+/-}$ mice and *Gabrb2*-CKO mice. **b, c** Quantification of ejection fraction (EF%) (**b**) and fractional shortening (FS%) (**c**) from *Cre*$^{+/-}$ mice and *Gabrb2*-CKO mice after 8-week tamoxifen induction. n = 10 mice for *Cre*$^{+/-}$ group and n = 11 mice for *Gabrb2*-CKO group. Data are shown as mean ± s.d.. P values were calculated using two-tailed unpaired student t test.
