# Supplementary material for: A GABAergic system in atrioventricular node pacemaker cells controls electrical conduction between the atria and ventricles
Source: Cell Res. 2024 Jun 7;34(8):556–71. doi: 10.1038/s41422-024-00980-x (PMC11291642; doi:10.1038/s41422-024-00980-x)
Supplement: Supplementary file 18 — Supplementary information, Fig. S18 [file 41422_2024_980_MOESM18_ESM.pdf]

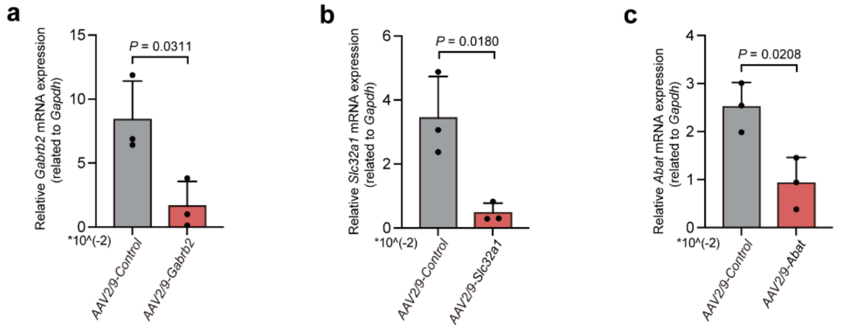

Supplementary information, Fig. S18 The expression levels of *Gabrb2*, *Slc32a1* and *Abat* are decreased in AAV2/9 knockdown virus-injected AVN tissues. Data are shown as mean ± s.d.. *P* values were calculated using two-tailed unpaired student t test. *n* = 3 mice per group.
