# Supplementary material for: A GABAergic system in atrioventricular node pacemaker cells controls electrical conduction between the atria and ventricles
Source: Cell Res. 2024 Jun 7;34(8):556–71. doi: 10.1038/s41422-024-00980-x (PMC11291642; doi:10.1038/s41422-024-00980-x)
Supplement: Supplementary file 20 — Supplementary information, Table S1 [file 41422_2024_980_MOESM20_ESM.pdf]

Supplementary information, Table S1 The primers used for genotype.

Gene Target	Sequences (5' to 3')
Hcn4-Cre	GCCTGCATTACCGGTCGATGC
	CAGGGTGTTATAAGCAATCCC
Rosa26-tdTamoto	AAGGGAGCTGCAGTGGAGTA
	CCGAAAATCTGTGGGAAGTC
	GGCATTAAAGCAGCGTATCC
	CTGTTCCTGTACGGCATGG
Gabrb2$^{flox/flox}$	CGCATTCTTTCTATGTGCTCCTG
	TCAAGACAGGGTTTCTCTGTGTAGC
