# Supplementary material for: A GABAergic system in atrioventricular node pacemaker cells controls electrical conduction between the atria and ventricles
Source: Cell Res. 2024 Jun 7;34(8):556–71. doi: 10.1038/s41422-024-00980-x (PMC11291642; doi:10.1038/s41422-024-00980-x)
Supplement: Supplementary file 21 — Supplementary information, Table S2 [file 41422_2024_980_MOESM21_ESM.pdf]

Supplementary information, Table S2 The primers used for qPCR.

Gene (Mus)	Forward Primer (5' to 3')	Reverse Primer (5' to 3')
Slc6a1	CCGTGAACTCTTCATTGCTGCC	ACCAGGAACAGCAAGCTCATGC
Slc6a13	ATACACGCCACTGACCTACAACAAG	CAGGAATGCAGATCATGGAGGACAG
Slc6a12	CCTGAACAGTGCTACCAGCTTC	GGCATCATGGTGACAGCTTTGG
Slc32a1	GGCTGGAACGTGACAAATGCCA	TACAGGCACGCGATGAGGATCT
Slc17a7	TGGCTGTGTCATCTTCGTGAGG	TTGCCAGCCGACTCCGTTCTAA
Slc6a11	GGTTTGCCATCTTCTCAGTCCTG	GGGCATCATAGTGACAGCCTTG
Slc17a6	CCTATGCTGGAGCAGTCATTGC	GGCTCTCATAAGACACCAGAAGC
Slc1a3	GCGATTGGTCGCGGTGATAATG	CGACAATGACTGTCACGGTGTAC
Gabra1	TTCACAAGAATTTTGGACCGAC	CCTTCCAACTTTGACGGAAAAA
Gabra2	CTCTCCCAAGTGTCATTCTGGC	CGAGCACTGATGCTCAAGGTTG
Gabra3	GTCATCCAGACCTACTTGCCATG	CTGGCACTGATACTCAAGGTGG
Gabra4	GAAGTCAGTGGAGGTGCCAAAG	TCTGAGGTGGAAGTAAACCGTCA
Gabra5	CAATGGCTCCACCAAGTCTGTG	CAGGTGGAAGTGAGCAGTCATG
Gabra6	GGAAGGCTATGACAACCGTCTAC	CAGTCCAAGTCTGGCGGAAGAA
Gabrb1	GCGAGCAAACAAGACCAGAGTG	AGCCACTGGTCTCGTTCCTGAT
Gabrb2	CTAAGGCGGTATCCACTGGATG	CTACGATGGAGAACTGAGGAAGC
Gabrb3	AGCGTGCATGATGGACCTCAGA	TTTCCACGCCAGTGACAGCCTT
Gabrg1	CCTCTGTGGAAGTAGCTGATCC	CCATCCGTCTGCTCAGGTCAAA
Gabrg2	ATCACCACTCCCAACAGGATGC	GCAGGAGTGTTCATCCATTGGG
Gabrg3	ACTGACTGTGGTTCTGTCCTGG	GCAGAGACTTCCTGGCAATGGT
Gabre	GAAGCCTACTGTGGTCACTGTTG	GAAGACGCTCATCATACCAGGTC
Gabrq	GTTCAGAGGGAAGTCCGCAGTT	GTCGATGGTAGTCAGGACAAGG
Gabbr1	CGTGGGACTTTTCTATGAGACCG	GAACCAGTTGTCAGCATACCACC
Gabbr2	TACACAACCGCCTTTGGAGCCA	CCACAATCACGAGCAGCTTCTG
Gabrr1	CTGGACAGCATCTCAGAGGTTG	GCCATCAAAGGTCATGCTGAGG
Gabrr2	CAAACGCTCCTTCATCCACGAC	GTGGCTGAAGTCCATGTTGCAC
Gad1	CGCTTGGCTTTGGAACCGACAA	GAATGCTCCGTAAACAGTCGTGC
Gad2	CCTTGCAGTGTTCAGCTCTCCT	GCCTTGTCTCCTGTGTCATAGG
Abat	TGCTCCATTCCCACGGCTGAAA	GATTCCAGCCACCGTTCTCTTC
Aldh5a1	CCTTTACTGGCTCAACAGCAACG	CTGGTCTACGTTGGCACTGTCA
Gapdh	AGGTCGGTGTGAACGGATTTG	TGTAGACCATGTAGTTGAGGTCA

Gene (Rat)	Forward Primer (5' to 3')	Reverse Primer (5' to 3')
Gabrb2	TGACTGATGTGAACGCCATTGATC	AGCCAATAGACGATGTTGAAGAAGG
Slc32a1	TCGTCATCGGCGGCATCTG	TCCTCTGCGTTGGTTCGGTAG
Abat	GAGCCCATCCAGTCCGAAGG	AGTCTGAACCTCGTCCACCAAG
Gapdh	ATGGGGAAGGTGAAGGTCG	GGGGTCATTGATGGCAACA
